# Transformational Catalytic Innovation Supports CO_2_ Resource Utilization: Bridging Sustainability and Industrial Applications

**DOI:** 10.1002/open.70222

**Published:** 2026-05-14

**Authors:** Xuexue Pan, Yi Zhao, Dinmukhambet Baimbetov, Yevgeniy Muralev, Samal Syrlybekkyzy, Berik Bakhytzhanovich Akhmetov, Saman Khosravi Haji, Qamar Abbas

**Affiliations:** ^1^ Zhongshan Advanced New Functional Materials Engineering Technology Research Center Laboratory of Advanced Functional Materials Zhongshan Polytechnic Zhongshan China; ^2^ Leibniz Institute for Catalysis Rostock Germany; ^3^ Yessenov University Aktau Kazakhstan; ^4^ Faculty of Chemical Technology Poznan University of Technology Poznan Poland

**Keywords:** CO_2_ conversion, electrocatalysis, heterogeneous catalysis, single‐atom catalysts, sustainable chemistry

## Abstract

This article presents a systematic review and establishes a fundamental distinction between two primary technological pathways for catalytic carbon dioxide (CO_2_) conversion: thermocatalytic conversion and electrocatalytic reduction. Within thermocatalytic pathway, the application of heterogeneous catalysts and homogeneous molecular catalysts in reactions such as CO_2_ hydrogenation and dry reforming has been discussed. It details strategies for optimizing CO_2_ activation and selective hydrogenation through the design of active sites (e.g., metal–support interfaces, oxygen vacancies) and ligand environments, while examining prevalent catalyst deactivation mechanisms. For the electrocatalytic pathway, the focus is on renewable energy‐driven electrochemical CO_2_ reduction. The article summarizes the design of key electrode materials, highlighting how nanostructuring, alloying, and management of the local reaction microenvironment can achieve high selectivity toward either C1 or C2+ products. Advances in device engineering, such as membrane electrode assemblies, are also discussed. Furthermore, the unique role of single‐atom catalysts as a bridge between homogeneous and heterogeneous systems, demonstrating advantages across both pathways, has been elucidated. Finally, the article contrasts the common and distinct challenges facing the industrialization of these two routes and concludes by underscoring the importance of multidisciplinary integration in designing next‐generation high‐performance catalysts to accelerate the commercialization of CO_2_ conversion technologies.

## Graphical abstract

Thermocatalytic and electrochemical catalytic conversion/reduction of CO_2_ offer unique advantages yet face common issues at the electrode/electrolyte interface and toward process optimization and industrialization. This review discusses integrated strategies focused on long‐term catalyst stability and enhanced energy efficiency for achieving economic viability.

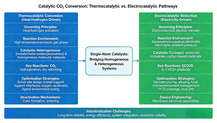

## 1 Introduction

Since the industrial revolution, the extensive use of fossil fuels worldwide has led to the atmospheric concentration of carbon dioxide (CO_2_) reaching historically high levels, triggering a series of severe climate change issues, such as global warming and ocean acidification, posing fundamental threats to the sustainability of Earth's ecosystems and human socioeconomic development. To address this challenge, it is imperative to reduce emissions at the source and actively explore strategies for carbon recycling. Currently, the primary CO_2_ management strategies include Carbon Capture and Storage (CCS) and Carbon Capture, Utilization, and Storage (CCUS). Among these, the latter is particularly favored because it not only reduces emissions but also transforms CO_2_ into economically valuable carbon resources, making it recognized as one of the key technological pathways to achieving the “carbon neutrality” goal.

Against this backdrop, catalytic CO_2_ conversion technology demonstrates significant application potential. As a core method of chemical transformation, catalysis can effectively lower reaction barriers and guide the thermodynamically stable and inert CO_2_ molecules to be selectively converted into energy‐rich fuels (such as methane, methanol, and higher hydrocarbons) or high‐value‐added chemicals (such as syngas, formic acid, and olefins) [1, 2]. This process simulates and accelerates the natural carbon cycle, aiming to create an artificial ’carbon closed loop’ that reduces net greenhouse gas emissions while generating new economic value, thereby achieving a win–win situation for both environmental and economic benefits.

However, the chemical inertness of CO_2_ molecules and the complexity of their reduction pathways place extremely high demands on catalytic technology. To achieve efficient and highly selective CO_2_ conversion, the scientific community has developed and studied various catalytic systems. Traditional heterogeneous catalytic systems, with the advantage of easy catalyst separation and recovery, dominate large‐scale continuous industrial production. However, their active site structures are uneven, and the reaction mechanisms are complex, posing challenges in controlling product selectivity [3, 4]. In contrast, homogeneous catalytic systems have well‐defined and uniform active center structures, allowing precise control over reaction pathways through molecular design, thereby achieving extremely high product selectivity. However, the recovery and recycling of the catalyst remain a bottleneck for their practical application [5, 6, 7]. In recent years, the rise of single‐atom catalysts has cleverly combined the "single active site" advantage of homogeneous catalysis with the "stability and easy separation" characteristics of heterogeneous catalysis, providing a new platform for achieving highly active and selective CO_2_ conversion [8]. In addition, the electrocatalytic CO_2_ reduction technology driven by electricity generated from renewable energy sources (such as solar and wind energy) can convert CO_2_ into chemicals under mild conditions and directly couple carbon energy with renewable energy, making it a highly promising low‐carbon or even zero‐carbon conversion pathway [9, 10, 11].

**FIGURE 1:**
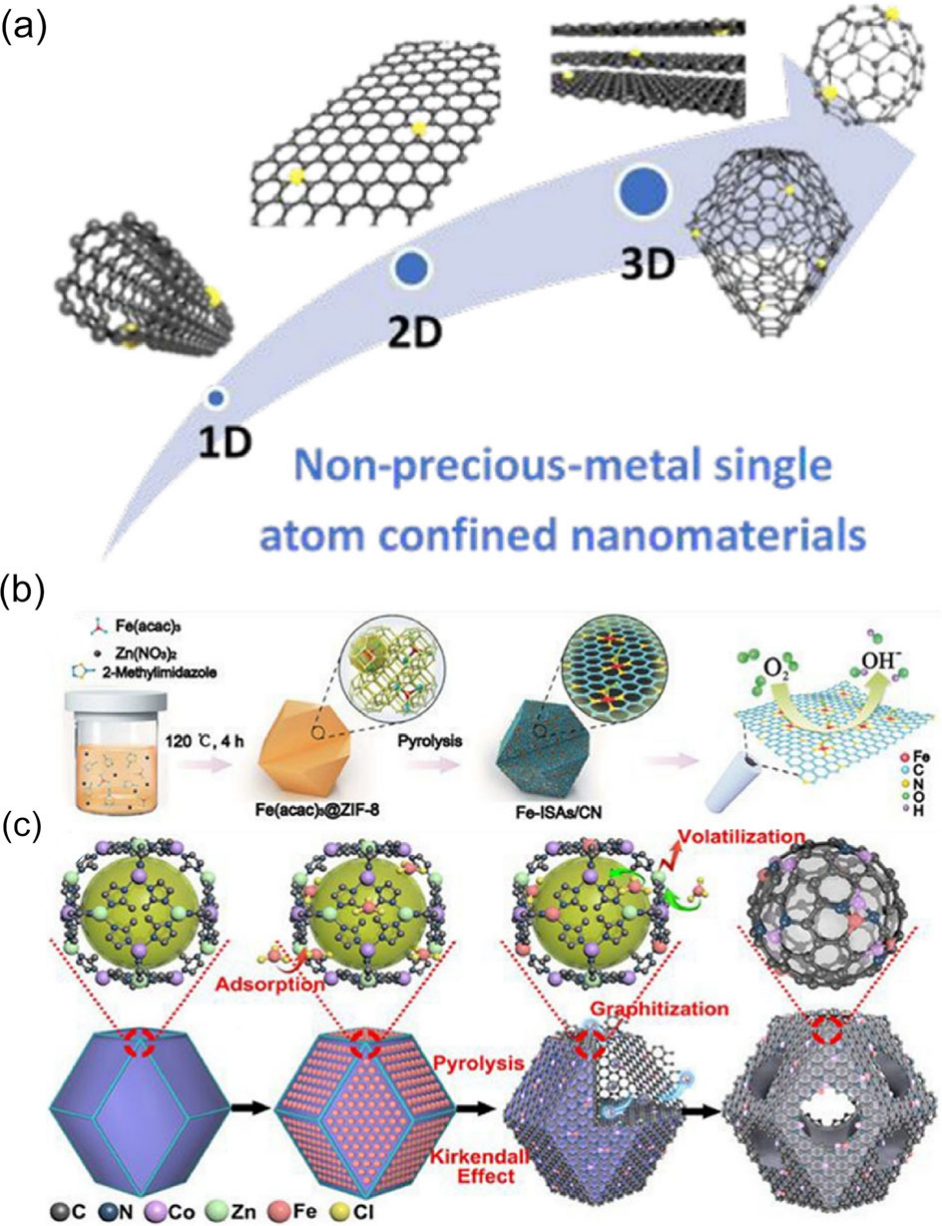
(a) Different structural dimensional supports confining single atoms [12]. (b) The formation of Fe‐ISAs/CN [12]. (c) The preparation of (Fe, Co)/N‐C [12]. Reproduced with permission. Adapted from [12]. Copyright 2020, Royal Society of Chemistry.

To construct a clear and logically coherent framework that prevents conceptual confusion, this review is structured around a two‐dimensional “CO_2_ Catalytic Conversion Technology Matrix” (Figure 1) [12]. The primary dimension is the energy input form, which fundamentally distinguishes two major technological pathways: (i) Thermocatalytic conversion, driven by thermal energy (often coupled with hydrogen), and (ii) Electrocatalytic reduction, driven by electrical energy [13]. These pathways operate under distinct principles (thermodynamics/kinetics vs. electrochemistry), conditions (high temperature/pressure vs. ambient aqueous), and performance descriptors (e.g., turnover frequency vs. Faradaic efficiency) [14]. The secondary dimension is the catalyst state, encompassing heterogeneous, homogeneous, and single‐atom systems, each offering unique advantages in active site design and control [15].

Guided by this matrix, this review will first conduct an in‐depth, parallel discussion of the two primary energy pathways [16]. For the thermocatalytic route, the design, mechanisms, and challenges of heterogeneous and homogeneous catalysts in reactions like CO_2_ hydrogenation will be examined [17]. For the electrocatalytic route, the focus will be placed on electrode materials, interfacial engineering, and device design for electrochemical CO_2_ reduction [18]. The role of single‐atom catalysts, which bridge concepts across catalyst states and demonstrate applicability in both energy pathways, will be discussed as a unifying frontier [19]. This structured approach aims not merely to catalog advances, but to compare the strengths, scientific principles, and applicability boundaries of different pathways, thereby providing clear logic and deeper insights.

This review aims to systematically summarize the latest research progress on the aforementioned catalytic technologies in the field of CO_2_ utilization and thoroughly explore the opportunities and challenges they face in scaling up to industrial applications. The article will first provide a detailed review of heterogeneous catalysis (including metal‐based, metal oxide, and perovskite catalysts), covering design strategies, reaction mechanisms, and performance optimization approaches. It will then analyze the unique advantages of homogeneous catalytic systems (particularly molecular catalysts) in precisely controlling product selectivity, along with the challenges related to their stability and recyclability. Next, the review will focus on single‐atom catalysts, an emerging frontier, discussing their distinct properties, application examples, and development directions. Furthermore, it will provide an outlook on reaction mechanism studies and new material development in the important trend of electrochemical CO_2_ reduction. Ultimately, the article will explore the primary challenges in scaling up catalytic CO_2_ conversion technologies from the laboratory to industrial scale, encompassing scalability, economic viability, and system integration. It will offer perspectives on future research directions, aiming to provide valuable insights for the development of next‐generation, efficient, stable, and scalable CO_2_ catalytic conversion technologies.

## 2 Multiphasic Catalysis

### 2.1 Background and Significance

As the primary source of greenhouse gases, CO_2_'s high thermodynamic stability makes direct conversion extremely costly [20]. Multiphasic (solid) catalysts, due to their ease of separation, suitability for continuous production, and long‐term operational durability, have become the core technological pathway for large‐scale industrial CO_2_ utilization [4, 21]. Driven by the transformation of the energy structure and the goal of achieving carbon neutrality, the design of solid catalysts with high activity, selectivity, and excellent stability has become a research hotspot in recent years.

### 2.2 Key Design Principles

The general design principles outlined in Table 1 above are foundational for catalyst development [15]; however, their specific interpretation and implementation are critically dependent on the targeted conversion pathway [15]. In thermocatalytic reactions (e.g., CO_2_ hydrogenation), the “synergistic adsorption” primarily refers to the co‐adsorption and activation of CO_2_ and H_2_ molecules on adjacent sites [25]. Strategies like creating metal–oxide interfaces or oxygen vacancies are employed to facilitate the heterolytic dissociation of H_2_ and the subsequent hydrogenation of activated CO_2_ intermediates [25]. Conversely, in electrocatalytic CO_2_ reduction, the “synergistic adsorption” emphasizes the coupled transfer of electrons and protons (H^+^) to the adsorbed CO_2_ molecule [26]. Here, the “metal coordination environment” and engineered vacancies often aim to optimize the binding strength of key electrochemical intermediates (like *COOH or *CO) and regulate the local proton availability to suppress the competing hydrogen evolution reaction [27]. Similarly, while “bimetallic synergy” in thermocatalysis often fine‐tunes the surface chemistry for specific C—H or C—O bond formation steps, in electrocatalysis it is frequently designed to control the coverage and coupling probability of *CO intermediates for multi‐carbon product formation [28]. This pathway‐dependent context is essential for the rational design of high‐performance catalysts.

**TABLE 1:** General design strategies for efficient CO_2_ conversion catalysts.

Goal	Key factor	Typical regulatory measures	Literature
High activity	CO_2_ activation and the synergistic adsorption of hydrogen/electrons	Metal oxide interfaces, oxygen vacancies, and metal coordination environment	[22]
High selectivity	Control of intermediate adsorption/desorption pathways	Bimetallic synergy, surface crystal facet selection, ligand/additive modulation	[23]
Excellent stability	Inhibit metal sintering, carbon deposition, and phase transformation	Nanometer size confinement, strong metal–support interaction, antioxidant/anti‐sulfidation materials	[24]

### 2.3 Mainstream Catalyst System

Precious metal‐based systems (such as Ru, Pd, and Pt) exhibit high catalytic activity in CO_2_ hydrogenation to produce methanol and methane; however, their application is limited by high costs [29]. Research shows that through Pd–Zn alloyed structures and the regulation of chloride ions, the Pd/ZnO system can significantly enhance the selectivity of methanol [30]. Meanwhile, non‐precious metals and their carbides or nitrides (such as Fe, Ni, Co, and Mo_2_N) also exhibit excellent durability in high‐temperature reverse water–gas shift (RWGS) reactions [31]. Recent studies have further indicated that constructing a sub‐nanometer MoO_*x*_ layer on the surface of Mo_2_N enables the direct cleavage of C=O double bonds, thereby significantly enhancing the efficiency of CO production [32]. In terms of single‐atom and dual‐atom catalysts, atomically dispersed metals such as Au, Fe, Pd, and Co induce the formation of abundant oxygen vacancies through strong interactions with oxide supports, effectively enhancing the activation capability of CO_2_. This enables such catalysts to exhibit both high selectivity and good anti‐sintering performance in low‐temperature CO_2_ reduction [33, 34]. In addition, oxide composite systems (such as In_2_O_3_–ZrO_2_, CeO_2_–Cu, ZnO–ZrO_2_) enhance the density of surface active sites through oxygen vacancy engineering, promoting the formate pathway in the methanol synthesis process while effectively suppressing side reactions such as carbon deposition [35]. On the other hand, carbon‐based materials such as nitrogen‐doped carbon or metal catalysts supported on graphene can enhance the activation ability of H_2_ through electron transfer regulation, making them suitable for electrochemical CO_2_ reduction reactions at high current densities [36, 37].

#### 2.3.1 Microscopic Insights Into Reaction Mechanisms and Active Sites

A deep understanding of the reaction mechanisms at working conditions is pivotal for rational catalyst design [38]. For the mainstream systems discussed above, advanced in situ and operando characterization techniques have been instrumental in elucidating the nature of active sites and the dynamic evolution of surface intermediates in thermocatalytic CO_2_ hydrogenation [39].

For metal‐based systems (e.g., Pd/ZnO, Cu‐based catalysts), in situ diffuse reflectance infrared Fourier transform spectroscopy (DRIFTS) and ambient pressure X‐ray photoelectron spectroscopy (AP‐XPS) studies have revealed that the metal–oxide interface often serves as the crucial site for CO_2_ activation [40]. On Cu–ZnO catalysts, for instance, in situ DRIFTS has identified formate (HCOO) and methoxy (OCH_3_) species as key intermediates in the pathway to methanol [41]. The synergy at the Cu–ZnO interface facilitates H_2_ dissociation on Cu and spillover to adjacent ZnO sites, where CO_2_ is activated, promoting the hydrogenation sequence via the formate route [42, 43].

In oxide and perovskite systems, the role of oxygen vacancies (OVs) as primary active sites has been directly corroborated. In situ electron paramagnetic resonance (EPR) and X‐ray absorption spectroscopy (XAS) demonstrate that OVs on surfaces of CeO_2_ or In_2_O_3_‐based catalysts act as electron donors, promoting the heterolytic dissociation of H_2_ and the formation of reactive hydrogen species (e.g., hydrides or hydroxyl groups) [44]. These species subsequently hydrogenate adsorbed CO_2_ into formate or carbonate intermediates. The concentration and mobility of these vacancies, often tunable by doping or reduction pretreatment, correlate directly with catalyst activity and selectivity toward methanol or CO [45, 46].

For single‐atom catalysts (SACs), combined computational studies and in situ XAS have provided atomic‐level insights [47]. Isolated metal atoms (e.g., Pt_1_, Pd_1_) on oxide supports can activate CO_2_ via a unique metal–support interfacial bonding, differing from the mechanisms on nanoparticles [48]. Their exceptional selectivity often stems from suppressing side reactions like CO dissociation or over‐hydrogenation to methane, which require ensemble sites unavailable on isolated atoms [49].

These mechanistic insights establish clear structure–activity relationships: the catalytic performance in thermocatalytic hydrogenation is governed by the synergistic function of sites for H_2_ activation (often metal sites) and CO_2_ activation (often oxide or vacancy sites), and the relative stability of C_1_ oxygenates (formate, methoxy) versus CH_*x*_ species dictates the selectivity toward oxygenates (methanol) versus hydrocarbons (methane) [50]. This foundational understanding directly informs the design principles outlined in Section 2.2, moving catalyst development from trial‐and‐error to a more predictive science.

### 2.4 Regulation Strategies for Surfactant Active Sites

The regulation strategies summarized in Table 2 are pivotal for enhancing catalyst performance; however, their operational mechanism and primary objective are intrinsically linked to the specific CO_2_ conversion pathway. In thermocatalytic hydrogenation, the ultimate goal of these strategies is to orchestrate the efficient activation and co‐adsorption of CO_2_ and H_2_, steering the reaction along desired hydrogenation pathways [25]. For instance, creating a metal–oxide interface (e.g., Pd/ZnO) often aims to establish a bifunctional site where H_2_ dissociates on the metal and spilled‐over hydrogen species react with CO_2_ activated at the oxide periphery, promoting formate intermediates for methanol synthesis [53]. Similarly, oxygen vacancies in oxides primarily serve as sites for activating the inert CO_2_ molecule and sometimes as reservoirs for active hydrogen [54]. Bimetallic synergy is frequently designed to achieve a division of labor, where one metal facilitates H_2_ dissociation and the other optimizes CO_2_ adsorption or intermediate hydrogenation [55].

**TABLE 2:** Catalyst modification strategies for enhancing CO_2_ conversion performance.

Regulatory measures	Mechanism of action	Representative study	Literature
Metal‐oxide interface	Form an electron transfer layer, generating strong adsorption of CO_2_ and H, promoting the formation of formate intermediates.	Pd/ZnO, In_2_O_3_–ZrO_2_	[51]
Oxygen vacancy/defect engineering	Provide additional electron donors to lower the CO_2_ activation energy barrier.	Au–Fe single atoms, MoO_*x*_ on Mo_2_N surface	[52]
Metal coordination environment	Adjust the occupancy of the metal's d‐orbitals through ligands or additives to alter the adsorption geometry.	Pd‐Cl‐Zn alloying	[51]
Size/Morphology control	The Nanometer scale increases specific surface area and suppresses metal sintering.	Mo_2_N covered with sub‐nanometer MoO_x_	[52]
Bimetallic synergy	Division of labor in H_2_ activation and CO_2_ adsorption through electronic/geometric effects	Pd–Cu, In‐Pd bimetallic systems	[51]

**In contrast, for electrocatalytic CO_2_ reduction, the same categories of strategies are employed but with a different focus managing the adsorption strength of key electro‐generated intermediates (like *COOH, *CO) and controlling the coupled proton‐electron transfer kinetics to outcompete the hydrogen evolution reaction (HER) [56]. Here, a metal–oxide interface or oxygen vacancies might be engineered to modulate the d‐band center of the metal site, thereby optimizing *CO binding energy to favor either its desorption as CO or its further reduction/coupling [57]. Size/morphology control and nanostructuring are crucial for exposing high‐index facets that stabilize *CO at high coverage, a prerequisite for C—C coupling on Cu. Bimetallic synergy in this context is often used to tailor the electronic structure for a specific intermediate (e.g., COOH for CO production) or to create tandem systems where one metal generates a high local concentration of CO for subsequent reduction on a neighboring site [58]. Therefore, a clear recognition of this pathway‐dependent context is essential for the targeted application of these fundamental regulation strategies.

### 2.5 Representative Latest Progress

Among the representative advancements from 2024 to 2025, various catalytic systems achieved significant breakthroughs in CO_2_ conversion performance. Precious metal systems such as Ru, Pd, and Pt can achieve over 30% methanol conversion under high‐pressure hydrogenation conditions. Moreover, by precisely designing metal–oxide interactions (e.g., Pd/ZnO), it is possible to maintain high reaction selectivity while effectively reducing the amount of precious metal used [59, 60]. On the other hand, innovations in non‐precious metal systems are equally remarkable. The sub‐nanostructured MoO_x_/Mo_2_N catalyst increased CO yield by 2.5 times through direct cleavage of the C=O double bond at temperatures above 600°C, offering a new strategy for the high‐temperature reverse water–gas shift reaction [61, 62]. In the field of atomic‐level catalysis, single‐atom Au–Fe catalysts exhibit 85% CO selectivity in the electrochemical reduction of CO_2_. They can maintain stable activity over a continuous 100‐hour operation, fully demonstrating their exceptional durability [63].

Additionally, significant progress has been made in oxide composite systems. Studies have found that in the In_2_O_3_–ZrO_2_ system, when the optimal indium loading is 9 wt%, a methanol yield of up to 12.3 mmol g^−1 ^h^−1^ can be achieved. Mechanistic studies have confirmed that this excellent performance is positively correlated with the concentration of oxygen vacancies and the stability of formate intermediates [64].

### 2.6 Challenges and Prospects of Industrialization

Although CO_2_ catalytic conversion technology has made significant progress, its industrialization process still faces multiple challenges and opportunities. First, cost and scalability are the core limiting factors, especially the high cost of precious metal catalysts. Future development should focus on economical alternatives such as non‐precious metals, alloys, and single‐atom catalysts (Figure 1a–c) [12, 65]. Second, the long‐term stability of the catalyst is crucial. Metal sintering and carbon deposition at high temperatures remain the primary mechanisms leading to deactivation, which necessitates enhancing the metal–support interaction and designing defect self‐healing structures to extend its lifespan [66]. At the same time, precise control of reaction selectivity is also crucial. The problem of by‐products (such as CO and CH_4_), which is common in heterogeneous catalytic systems, needs to be addressed by finely tuning the electronic structure of active sites to control the reaction pathways [67]. From a systems perspective, process integration is an essential path to promoting carbon neutrality, which involves efficiently coupling CO_2_ capture, renewable energy‐based hydrogen production, and catalytic conversion processes to build a full‐chain carbon‐neutral system [68]. Finally, intelligent characterization and machine learning are becoming powerful tools for overcoming research and development bottlenecks. By combining in situ/real‐time characterization techniques with AI‐driven materials screening, the discovery of new catalysts and the in‐depth analysis of reaction mechanisms will be greatly accelerated [69].

The core value of multiphase catalysis in CO_2_ conversion lies in efficient activation, controllable selectivity, and industrial durability [70]. In recent years, strategies such as metal–oxide interfaces, oxygen vacancy engineering, and single‐atom/bimetallic synergy have significantly enhanced the performance of catalysts [71]. The latest sub‐nanometer MoO_x_/Mo_2_N and Pd/ZnO systems have demonstrated breakthroughs in both high‐temperature reverse water–gas shift and low‐temperature hydrogenation. In the future, systematic research focusing on cost reduction, long‐term stability, and process integration will be key to achieving large‐scale CO_2_ utilization.

## 3 Metal‐Based Catalyst

Transition metals (such as Cu, Fe, Ni, Co, Pd) are pivotal across both major CO_2_ conversion pathways due to their partially filled d orbitals that enable effective interaction with CO_2_ molecules [72]. However, the design principles, performance targets, and underlying mechanisms for these metals differ fundamentally between thermocatalytic and electrocatalytic routes [73]. This chapter will primarily focus on the application and engineering of metal‐based catalysts within the electrocatalytic CO_2_ reduction (ECO_2_R) pathway, particularly highlighting strategies to achieve high selectivity towards multi‐carbon (C_2_^+^) products [74]. The role and design of metal catalysts in thermocatalytic hydrogenation (e.g., for methanol or methane synthesis) are discussed in depth within Sections 2 (Multiphasic Catalysis) and 4 (Metal Oxides and Perovskites) in the context of specific reactions and supports.

**FIGURE 2:**
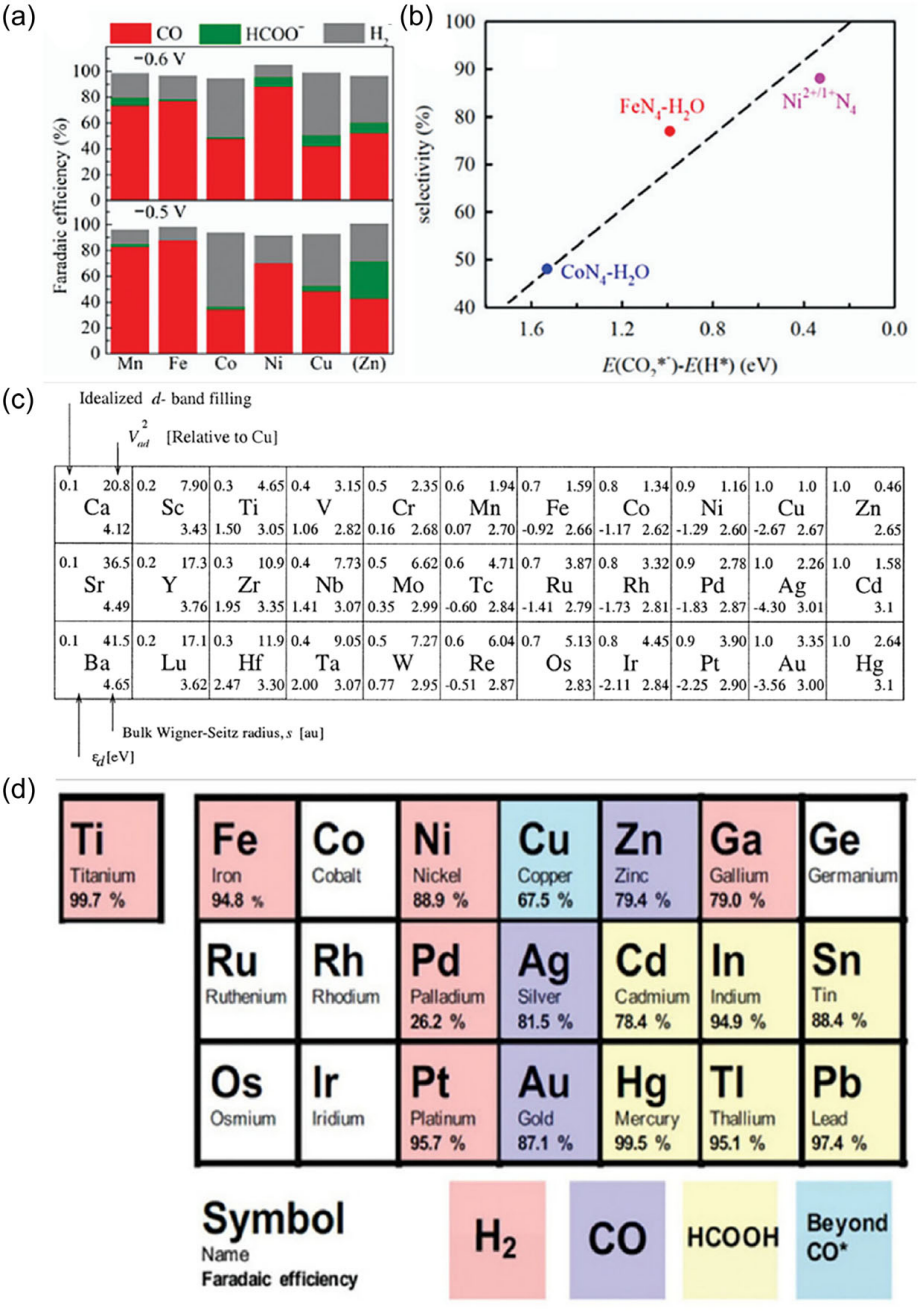
(a) FE at 0.6 V(up) and 0.5V (bottom) versus RHE obtained over the M—N—C catalysts (M = Mn, Fe, Co, Ni, and Cu) for 90 min electrolysis under CO_2_‐saturated 0.1 M KHCO_3_ aqueous electrolyte (pH 6.7) at room temperature [75]. (b) The experimental selectivity at 0.6 V versus RHE over Fe—N—C, Co—N—C, and Ni—N—C versus the DFT‐simulated E(*CO_2_) E(*H) [75]. Reproduced with permission. Adapted from [75]. Copyright 2020, Royal Society of Chemistry. (c) General trends in bonding strengths for transition metals [76]. Reproduced with permission. Adapted from [76]. Copyright 2020, Wiley‐VCH Verlag GmbH & Co. KGaA. (d) The different types of metal‐based CO_2_ reduction [77]. Reproduced with permission. Adapted from [77]. Copyright 2025, Wiley‐VCH GmbH.

Transition metals (such as Cu, Fe, Ni, Co, Pd), due to their partially filled d electron orbitals, can effectively activate CO_2_ molecules and are the most widely studied catalyst systems in CO_2_ hydrogenation (such as the Sabatier reaction, reverse water‐gas shift reaction, and Fischer–Tropsch synthesis) and electrochemical reduction (Figure 2a–c) [75, 76, 78, 79]. Transition metals (Cu, Fe, Ni, Co, Pd) can activate CO_2_ due to their unfilled d orbitals, which allow effective d–π* coupling with CO_2_. This electronic structure advantage makes them the most widely studied catalytic systems for CO_2_ hydrogenation and electroreduction. As shown in Figure 2a–c, the position of the d‐band center in different metals directly determines the product selectivity of M—N—C catalysts at 0.6 V (Figure 2a,b) as well as the relative bond strengths of M—CO and M—H (Figure 2c). From Mn to Cu, as the d‐band center shifts upward, CO adsorption becomes stronger while H adsorption becomes weaker. The experimentally measured FE(CO) and FE(H_2_) also show regular changes accordingly, thus fully linking the "d‐electronic structure–bond strength–catalytic selectivity" relationship [76]. Different metals exhibit varying adsorption strengths for reaction intermediates, leading to significant differences in product selectivity [80, 81]. For example, copper (Cu) is the only metal capable of efficiently electroreducing CO_2_ into multi‐carbon products (such as ethylene and ethanol), which is attributed to its moderate adsorption energy for CO intermediates, allowing the CO dimerization or coupling step for C—C bond formation to occur (Figure 2d) [77, 82, 83].

In recent years, precisely engineering the nanostructure to regulate catalyst surface properties has become a key strategy for enhancing performance. Specifically, in the field of electrochemical CO_2_ reduction, the selectivity and efficiency of C_2_^+^ products can be significantly improved through the design of nanostructures, alloying, and interactions with supports.

### 3.1 Nanostructuring and Exposing High‐Activity Sites

Nanostructuring is a key strategy for exposing highly active sites: by preparing Cu nanoparticles with high‐index facets (such as (210), (310)) through chemical etching or laser structuring, *CO adsorption can be significantly enhanced and C—C bond coupling promoted, thereby increasing the selectivity for C_2_^+^ products such as ethylene and ethanol [84]. Copper quantum dots with a size of approximately 5 nm achieved a Faradaic efficiency of 81.5% for ethylene at low potentials due to their extremely high *CO coverage and rapid CO—CHO coupling rate [85]. The Cu_2_O@CeO_*x*_ core–shell structure utilizes the abundant Cu/Ce interface to achieve electron transfer, which can both maintain the oxidation state of Cu_2_O and inhibit its aggregation, allowing the Faradaic efficiency for C_2_^+^ products to exceed 80% at 300 mA cm^−2^ [86, 87]. In addition, using a porous carbon carrier with a pore size of approximately 120 nm (CuHCS120) can increase the C_2_^+^ Faradaic efficiency to 46% by regulating the local CO concentration [88].

### 3.2 Alloy Effect With Synergy of Electronics and Geometry

**FIGURE 3:**
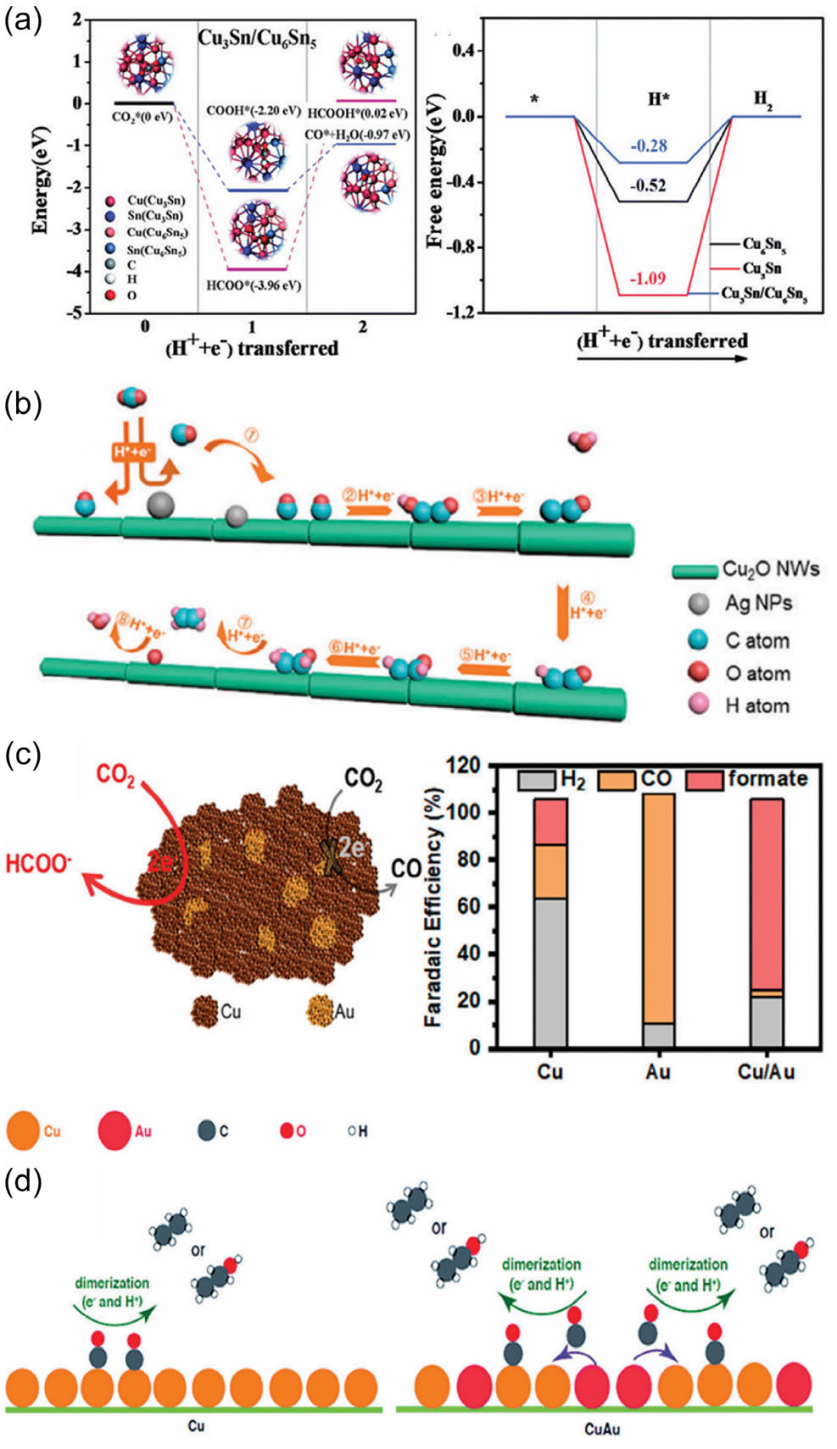
(a) The intermediate energy calculation diagram of CO and HCOOH on the surface of Cu_3_Sn/Cu_6_Sn_5_ heterostructure and the free energy diagram of HER on the three catalysts [90]. (b) Mechanism of CO_2_ reduction to C_2_H_4_ on Cu–Ag catalyst [90]. (c) CO_2_ reduction diagram on Cu/Au catalyst and FE distribution of CO_2_ products on Cu, Au, and Cu/Ag in 0.5 m KHCO_3_ aqueous solution saturated with CO_2_ [90]. (d) The formation mechanism of C_2_H_4_ and C_2_H_5_OH on Cu and CuAu [90]. Reproduced with permission. Adapted from [90]. Copyright 2024, Wiley‐VCH GmbH.

The alloy effect optimizes catalytic performance through the synergistic interaction of electronic and geometric structures. On atomically dispersed Cu‐Ag sites prepared by magnetron sputtering, Ag is responsible for supplying an abundant amount of CO. At the same time, the Cu–Ag interface can adjust the d‐band center, facilitating the cleavage of CO—CO—O intermediates and thereby achieving a 71% C_2_^+^ Faradaic efficiency at a high current density of 2.5 A cm^−2^ [89]. When 5.6 at% Ag forms a stable Ag/Cu^+^/Cu^0^ triple‐phase interface with Cu_2_O—Cu, it can significantly reduce the energy barrier for *CO—CO dimerization, achieving a C_2_^+^ efficiency of 76.5% at 1 A cm^−2^; Cu–Zn alloy in nitrogen‐doped porous carbon can provide additional CO generation sites, promoting the coupling of *CO and *CHO, resulting in an ethanol Faradaic efficiency of up to 77% [88]. In contrast, bimetallic systems such as Cu–Sn, Cu–Au, and Cu–Pd achieve a tunable distribution of products like ethylene, CO, and formic acid by inducing the selective adsorption of different intermediates (e.g., *COH, COOH, OCHO) (Figure 3a–d) [90]. As shown in Figure 3a, the Cu3Sn/Cu6Sn5 heterointerface preferentially promotes formic acid formation by calculating the difference in adsorption energies between CO and HCOOH and suppressing the HER free energy. In contrast, Figure 3b–d further present the formation pathways and FE distributions of CO_2_→C_2_H_4_ on Cu–Ag, CO_2_→CO on Cu/Au, and C_2_H_4_ and C_2_H_5_OH on CuAu, jointly revealing the synergistic regulation mechanism of bimetallic interface electronic effects and intermediate adsorption [90].

### 3.3 Carrier Interactions and Electron Transfer With Local Environment Regulation

The interaction between the support and the metal is crucial for regulating electron transfer and the local reaction environment. For metal oxide supports, when atomically dispersed Cu is loaded on oxygen‐deficient black TiO_2_, its oxygen vacancies can cooperatively provide hydrogen to stabilize the *CHO intermediate, resulting in a CH_4_ Faradaic efficiency of up to 63% [91]. The Cu/CeO_2_ interface enhances *CO adsorption through electron injection and enables a "volcano‐type" regulation of C_2_^+^ (62.6%) or CH_4_ (51.3%) via the CeO_2_ loading amount; the Cu/Co_3_O_4_ system utilizes strong metal–support interactions to enhance the synergistic activity of Cu^+^/Cu^0^. In terms of carbon‐based supports, amine‐modified single‐walled carbon nanotubes form a strong electronic coupling with Cu nanoparticles, which can increase CO and CHO coverage, resulting in a 66% Faradaic efficiency for C_2_^+^ products [92]. MOF‐derived or MOF‐encapsulated Cu‐based catalysts (such as Cu‐BTC, Cu–N_2_O_2_) can precisely regulate the Cu^0^/Cu^+^ ratio through controlled coordination environments, increasing the selectivity for C_2_^+^ products up to 79% [93]. In addition, various metal–metal oxide composite structures (such as Cu_2_O@CeO_*x*_ and Cu–CeO_*x*_ core–shell) have demonstrated tremendous potential for interface electron modulation to achieve long‐term stable operation at high current densities (>50 h) and high C_2_^+^ selectivity [94].

### 3.4 Comprehensive Regulation Approach

To achieve the efficient conversion of carbon dioxide to multi‐carbon products through electroreduction, current research has developed multidimensional, comprehensive regulation strategies, with the core design framework primarily covering four key directions: surface structure, electronic structure, interface, and support structure, as well as synergistic dual‐metal/single‐atom systems.

**FIGURE 4:**
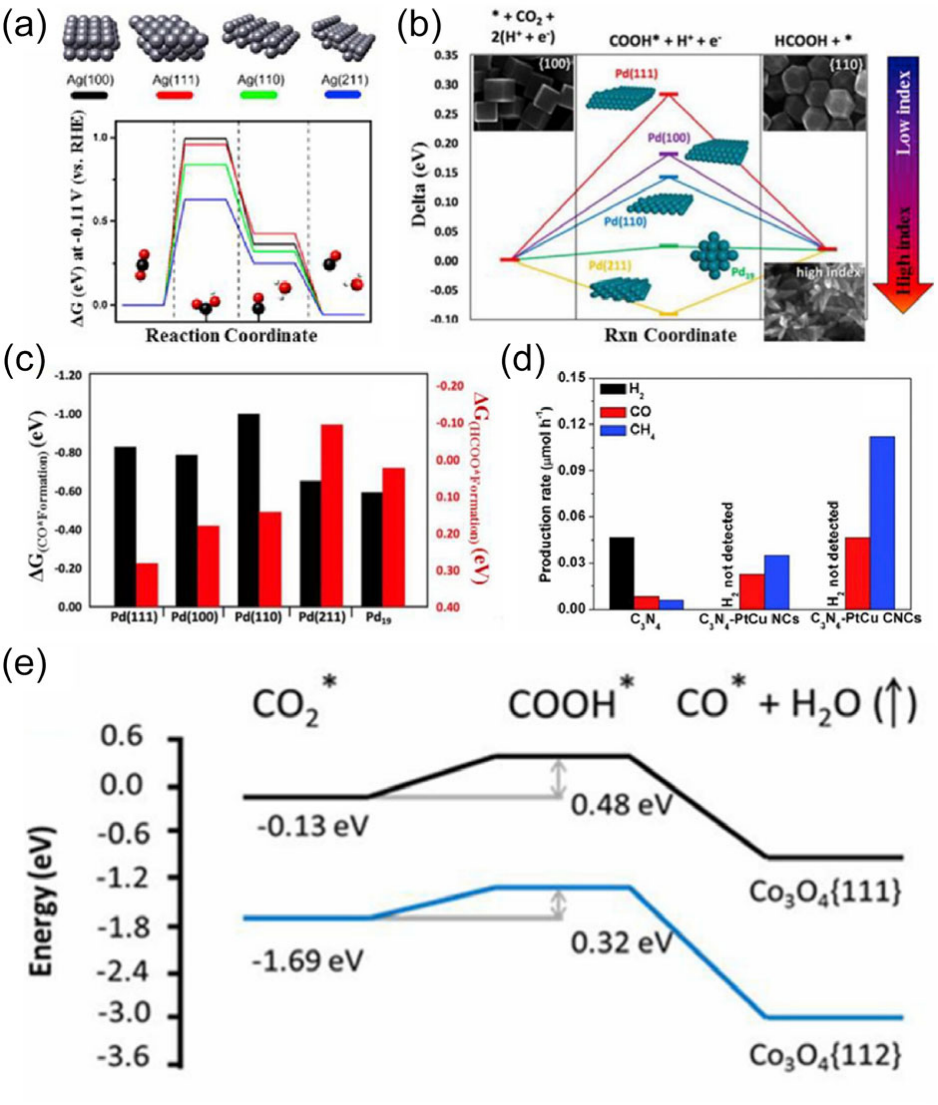
(a) Free‐energy diagrams for the electrochemical reduction of CO_2_ to CO on flat Ag(100) and Ag(111), as well as stepped Ag(211) and Ag(110) [95]. (b) Free‐energy diagrams for the CO_2_‐reduction reaction to formic acid and free energies of CHOO* and CO* intermediates on Pd single‐crystal surfaces and Pd19 cluster [95]. (c) Free energies of formation for HCOO* and CO* intermediates on Pd(111), Pd(100), Pd(110), Pd(211), and Pd19 cluster [95]. (d) Photocatalytic H_2_,CO, and CH_4_ evolution rates with the samples of C_3_N_4_ and C_3_N_4_‐PtCu NCs and C_3_N_4_‐PtCu CNCs during CO_2_‐reduction reaction [95]. (e) DFT calculation of adsorption and reduction of CO_2_ on Co_3_O_4_ surfaces [95]. Reproduced with permission. Adapted from [95]. Copyright 2020, Cell Press.

In terms of surface structure regulation, methods such as constructing high‐index crystal facets, introducing surface step defects, fabricating quantum dots, or applying pore confinement can effectively enhance the adsorption of *CO intermediates and increase their local concentration, thereby significantly accelerating the C–C coupling process (Figure 4a–e) [95]. Typical examples, such as Cu quantum dots with a size of about 5 nanometers and CuHCS120 materials with specific pore structures, both exhibit excellent performance [96]. As shown in Figure 4a, Ag(211) and Ag(110) stepped surfaces are more prone to CO accumulation because their CO adsorption energies are significantly lower than those of flat Ag(100) and Ag(111) surfaces. Figure 4b,c further shows that defects on Pd single crystals and Pd19 clusters similarly lower reaction barriers by stabilizing CHOO and CO intermediates. Figure 4d confirms that the porous/high‐curvature structure of C_3_N_4_‐PtCu nanocages (CNCs) simultaneously enhances the yields of H_2_, CO, and CH_4_ in photocatalytic CO_2_ reduction. In Figure 4e, the high‐index {112} facets of Co_3_O_4_ hexagonal plates significantly lower the free energy of the CO pathway due to strong COOH adsorption. Together, these findings reveal a general structure–activity relationship: "defects/high‐index facets → *CO accumulation → accelerated C—C coupling" [95].

In the realm of electronic structure regulation, strategies commonly employed include alloying (such as Cu–Ag, Cu–Zn, and Cu–Sn) or elemental doping (such as B and I) [97, 98]. This type of method can finely tune the position of the d‐band center of Cu, alter the competitive adsorption relationship between CO and H, and stabilize certain key reaction intermediates. For example, atomically dispersed Cu–Ag sites and B‐doped Cu catalysts fall into this category [51, 99].

Interface/support engineering focuses on constructing heterogeneous structures such as metal–oxide (e.g., TiO_2_, CeO_2_, Co_3_O_4_), carbon‐based functionalized materials, or MOF/COF composites. These interfaces can induce significant electron transfer, utilize oxygen vacancies (VOs) in oxides to provide hydrogen species, regulate local pH and CO_2_ concentrations, and stabilize highly active phases [100]. The representative Cu–TiO_2_–VO system and the ’volcano‐type’ activity relationship exhibited by the Cu/CeO_2_ interface both reflect the success of this strategy.

In addition, the design of synergistic bimetallic/single‐atom systems, such as Ag/Cu^+^/Cu^0^ biphasic interfaces, composites with metal phthalocyanines (CoPc, NiPc), or the construction of Cu–Ag single‐atom alloys, can achieve a multiple‐fold increase in CO production efficiency. The underlying mechanism typically involves CO spillover effects and the enhanced stability of key intermediates (such as *CO—CO—O), collectively driving the efficient formation of multi‐carbon products [101].

### 3.5 Research Frontiers and Prospects

**FIGURE 5:**
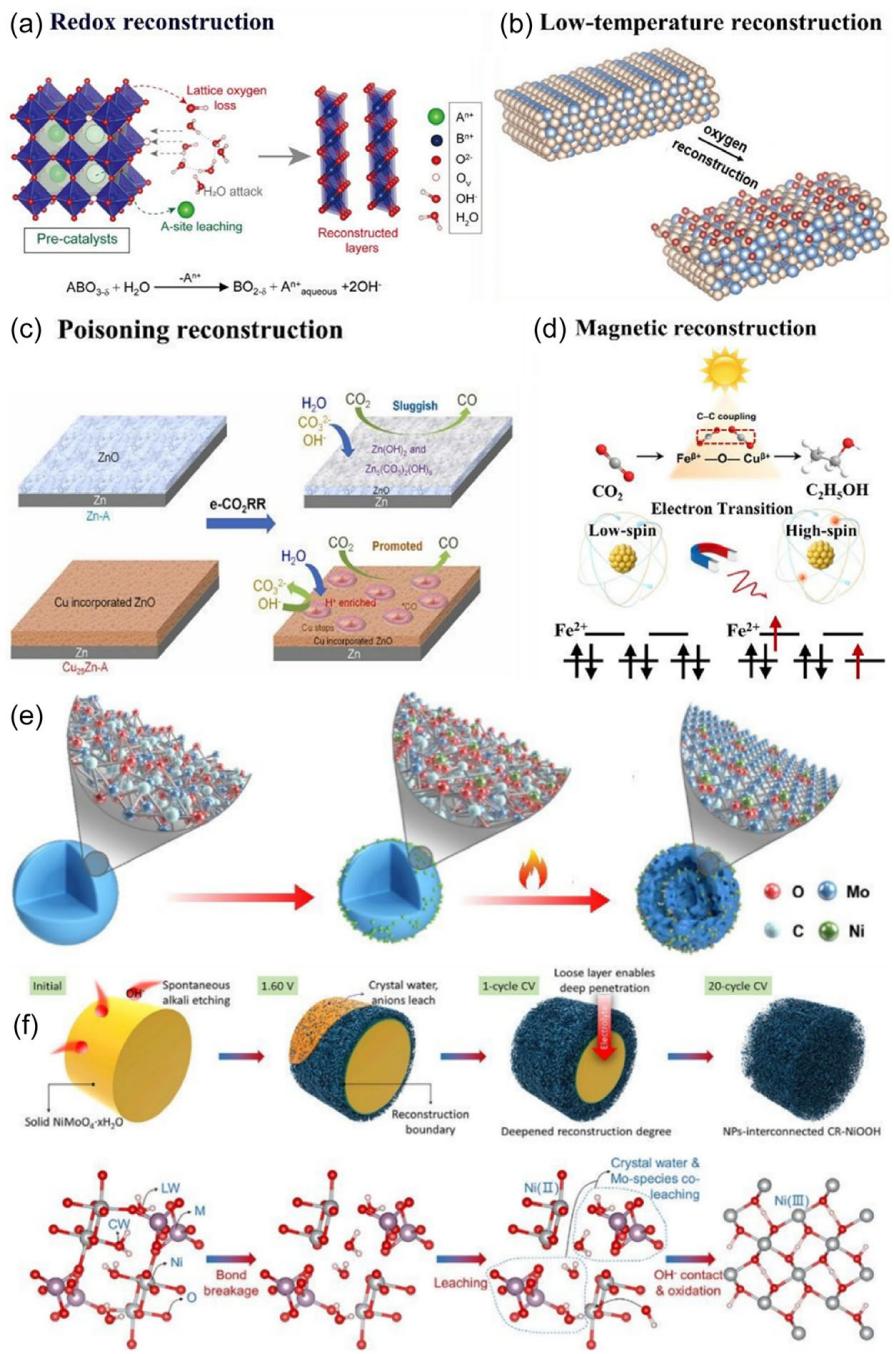
(a) A spontaneous chemical reaction process triggered by surface reconstruction [102]. (b) Low‐temperature oxidation reconstruction results in atomic rearrangements, introducing new catalytic sites [102]. (c) Poisoning reconstruction promotes the in situ growth of catalyst surfaces [102]. (d) The magnetic field alters the valence electron orbitals of metal atoms [102]. (e) During the thermal field, the structure and site of the catalyst are reconstructed [102]. (f) Geometric/phase changes, proposing the reconstruction mechanism from the perspective of crystal structure [102]. Reproduced with permission. Adapted from [102]. Copyright 2025, Royal Society of Chemistry.

To achieve the transition from fundamental research to industrial applications in the electroreduction of carbon dioxide to multi‐carbon products, current research frontiers and prospects are focusing on the following key directions:

The advanced application of in situ/real‐time characterization technology is key to revealing reaction mechanisms [102]. As shown in Figure 5a–f: surface reconstruction can spontaneously trigger chemical reactions (Figure 5a), low‐temperature oxidation induces atomic rearrangement and introduces new active sites (Figure 5b), poison adsorption drives in situ growth on the catalyst surface (Figure 5c), magnetic fields regulate the valence electron orbitals of metal atoms (Figure 5d), thermal fields alter the catalyst's geometric structure and site distribution (Figure 5e), while the schematic of geometric/phase changes from the perspective of crystal structure (Figure 5f) systematically outlines the "external field–reconstruction–activity" coupling mechanism, providing a panoramic framework for in situ analysis of catalytic evolution pathways [102]. By combining advanced in situ techniques such as Raman, FTIR, and XAS, researchers were able to directly capture the surface reconstruction process of catalysts and the dynamic behavior of key intermediates *CO under high current density operation, thus providing the most direct evidence that ’the active site is located at the reaction site’ [103]. At the theoretical level, multi‐scale simulations serve as a bridge between atomic‐level understanding and the prediction of microscopic processes [1]. Through a systematic approach from density functional theory (DFT) to microscopic kinetic Monte Carlo simulations, the impact of electronic structure modulation and interfacial charge transfer on the energy barriers of key coupling steps, such as *CO—*CHO and *CO—*CO, can be accurately assessed, providing a theoretical basis for catalyst design [104]. For practical applications, engineering validation in terms of high current density and durability is crucial. This requires verifying, in industrially relevant configurations such as membrane electrode assemblies (MEA) or zero‐gap electrolyzers, whether nanostructures, alloys, and support systems can maintain long‐term stable operation (over 100 h) at industrial current densities of ≥ 1 A cm^−2^, which is a key metric for assessing their commercialization potential [105]. Ultimately, the large‐scale preparation of catalysts must consider sustainable synthesis. Developing low‐energy, recyclable routes for preparing alloys and support materials (such as magnetron sputtering, in situ deposition, MOF‐directed pyrolysis, etc.) is an essential requirement for advancing this technology toward large‐scale production [106].

Through the synergistic design of nanostructuring, alloy effects, and support interactions, it is possible to precisely regulate the adsorption strength and electronic structure of transition metals such as Cu, Fe, Ni, Co, and Pd on key CO_2_ intermediates (*CO, *CHO, *CO—CO, etc.), thereby achieving high selectivity for products (ethylene, ethanol, methane, C_2_^+^ oxides, etc.) and stable operation at high current densities [78]. The latest experimental and theoretical work (2023–2025) has provided substantial empirical evidence, indicating the key pathways for further advancing CO_2_ hydrogenation/electrochemical reduction technologies in the future, particularly in in situ characterization, interface engineering, and industrial‐scale applications.

## 4 Metal Oxides and Perovskites

This section focuses on the application of metal oxides and perovskites within the thermocatalytic CO_2_ conversion pathway, specifically for reactions such as CO_2_ hydrogenation and dry reforming [107]. The discussion here centers on their function under thermal driving forces, distinct from their occasional role as supports or co‐catalysts in electrochemical systems [108]. A core theme is the pivotal role of oxygen vacancies (OVs) as active sites, whose primary function in thermocatalysis is to facilitate the adsorption/activation of CO_2_ and to promote hydrogen dissociation and spillover—a mechanism fundamentally geared towards thermal hydrogenation processes [46].

Metal oxides (such as TiO_2_, ZnO, CeO_2_) and structurally tunable perovskites (general formula ABO_3_, such as LaFeO_3_, SrTiO_3_) play an important role in thermocatalytic CO_2_ conversion, especially in hydrogenation or dry reforming reactions that require high‐temperature conditions. The catalytic activity of these materials is closely related to their surface oxygen vacancy concentration and electronic structure.

**FIGURE 6:**
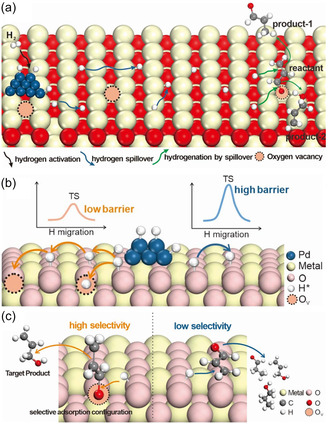
(a) **T**he effects of oxygen vacancies on the hydrogenation efficiency of an unsaturated substrate in the case of acrolein [110]. (b) Mechanism of oxygen vacancies affecting hydrogen spillover [110]. (c) Oxygen vacancies affecting hydrogenation by spillover [110]. Reproduced with permission. Adapted from [110]. Copyright 2025, Royal Society of Chemistry.

Oxygen vacancies (OVs) are key active sites in metal oxide catalysts for CO_2_ conversion [82, 109]. On the surfaces of materials such as TiO_2_, ZnO, and CeO_2_, oxygen vacancies provide under‐coordinated metal sites, promoting the chemical adsorption of CO_2_ in a bent O—C—O configuration, significantly reducing the bond energy of the C=O bond, thereby facilitating subsequent hydrogenation or cleavage reactions [82, 100]. In addition, oxygen vacancies can capture and store active H atoms, forming a hydrogen spillover pathway that enhances hydrogen dissociation and migration efficiency, thereby accelerating CO_2_ hydrogenation or dry reforming reactions (Figure 6a–c) [92, 110, 111]. Oxygen vacancies not only can capture and store active H atoms, constructing hydrogen spillover channels to enhance the efficiency of hydrogen dissociation and migration, thereby accelerating CO_2_ hydrogenation or dry reforming reactions; as shown in Figure 6a, in the model of crotonaldehyde hydrogenation, surface oxygen vacancies significantly improve hydrogenation efficiency by first activating H_2_, then transferring active hydrogen, and sequentially completing the hydrogenation steps of unsaturated substrates. Figure 6b,c further reveals how oxygen vacancies induce the hydrogen spillover mechanism, where the vacancy captures dissociated H* and directs its migration across the surface to the reaction sites, providing a continuous and efficient hydrogen source for deep CO_2_ reduction [110]. The concentration and distribution of oxygen vacancies can be regulated through reduction treatment, doping, or high‐temperature annealing. For example, the reversible Ce^3+^/Ce^4+^ redox pair in CeO_2_ enables the formation of a large number of oxygen vacancies at low temperatures, making it an efficient platform for CO_2_ activation [112].

The concentration and distribution of oxygen vacancies can be regulated through reduction treatment, doping, or high‐temperature annealing. For example, the reversible Ce^3+^/Ce^4+^ redox pair in CeO_2_ enables the formation of a large number of oxygen vacancies at low temperatures, making it an efficient platform for CO_2_ activation [113, 114]. By doping at the A site (such as La^3+^, Sr^2+^) or the B site (such as Fe^3+^, Ti^4+^), it is possible to regulate lattice distortion, oxygen vacancy formation energy, and electronic band structure, thereby precisely optimizing catalytic performance [115, 116]. It is particularly noteworthy that the introduction of A‐site vacancies can significantly increase the concentration of oxygen vacancies, reduce exciton binding energy, and enhance the separation efficiency of photogenerated electrons and holes [117]. For example, after the formation of Sr vacancies in Sr_2_TiFeO_6_, the selectivity of CO_2_ photoreduction to produce CH_4_ can reach 91%, with the yield increasing nearly fivefold [117]. In addition, during the high‐temperature reduction process, perovskites can precipitate metallic nanoparticles, such as Ni and Cu, which are in situ confined within the oxide framework, effectively inhibiting sintering and significantly improving long‐term stability in high‐temperature reactions, such as dry reforming [118].

In CO_2_ hydrogenation and dry reforming reactions, oxygen vacancy engineering offers multiple advantages: oxygen vacancies promote H_2_ dissociation and act as hydrogen reservoirs, thereby enhancing hydrogenation efficiency [82, 111]. Introducing oxygen vacancies into photothermal catalysts, such as TiO_2_, ZrO_2_, and In_2_O_3_, can achieve dry reforming at temperatures below 300°C, significantly reducing energy consumption [119]. The vacancy at position A can also modulate the electronic structure of the metal at position B, thereby optimizing the adsorption behavior of key intermediates and enhancing CH_4_ production activity [120]. In addition, in systems such as TiO_2_–Cu and ZnO–Cu, strong metal–support interactions are enhanced through oxygen vacancies, promoting the conversion of CO_2_ into methanol or methane [121, 122].

**FIGURE 7:**
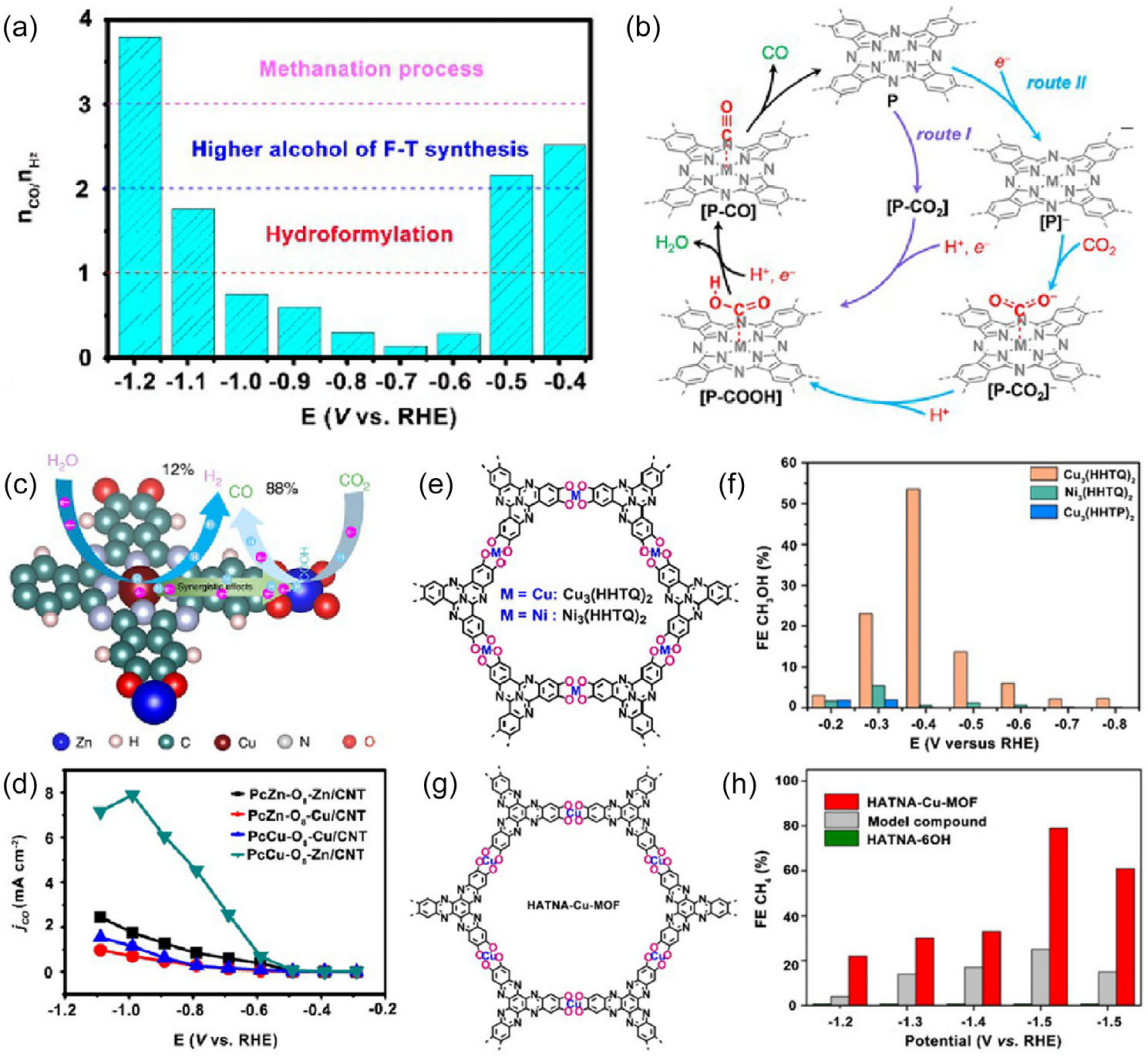
(a) Molar H_2_/CO ratio of PcCu‐O_8_‐Zn/CNT at different applied potentials [124]. (b) The proposed reaction mechanism of CoPc‐Cu‐O [124]. (c) HER and CO_2_RR process of PcCu‐O_8_‐Zn [124]. (d) Partial current density of CO for PcM‐O_8_‐M1/CNT (M/M1=Cu, Zn) in 0.1 M KHCO_3_ [124]. (e) Synthetic scheme of M_3_(HHTQ)_2_ (M = Cu, Ni) [124]. (f) FE of CH_3_OH (h) of Cu_3_(HHTQ)_2_, Ni_3_(HHTQ)_2_ and Cu_3_(HHTP)_2_ in 0.1 M KHCO_3_ [124]. (g) Synthetic scheme of HATNA‐Cu MOF [124]. (h) FE of CH_4_ for HATNA‐Cu, model compound, and HATNA‐6OH [124]. Reproduced with permission. Adapted from [124]. Copyright 2022, American Chemical Society.

To effectively design high‐performance catalysts, it is necessary to integrate various strategies: controllable generation of oxygen vacancies can be achieved through reduction annealing, chemical reduction, or doping (such as Nb^5+^, W^6+^) [123]. Regulating the electronic structure and adsorption properties through A/B site co‐doping (Figure 7a–h) [124]. Utilizing A/B site co‐doping can precisely regulate the electronic structure and adsorption properties, thereby achieving efficient control over the selectivity of CO_2_ reduction products. As shown in Figure 7a–d, PcCu‐O_8_‐Zn/CNT adjusts the adsorption strength of CO and H through the synergistic effect of Cu/Zn bimetallic sites, maintaining the H_2_/CO molar ratio at ˜1 over a wide potential window, featuring both a high partial current density for CO and the ability to suppress HER. Figure 7e–h further reveals that in Cu_3_(HHTQ)_2_ and HATNA‐Cu MOFs, the Cu–N_4_/O_4_ coordination environment optimizes the stability of CH_3_O and CH_4_ intermediates through A/B site electronic coupling, significantly enhancing the Faradaic efficiencies for methanol and methane. Together, these results demonstrate the general design principle of "A/B site co‐doping → fine‐tuning of adsorption energy → switching of product pathways" [124]. Metal nanoparticles are confined on the perovskite framework using impregnation‐reduction or in situ deposition methods to prevent high‐temperature sintering [125]. It is also possible to combine light absorption enhancement techniques with oxygen vacancy engineering to construct a photothermal/photocatalytic coupling system, enabling efficient CO_2_ conversion at low temperatures [126].

Despite significant progress in oxygen vacancy engineering, many challenges remain. It is necessary to use techniques such as in situ XAS, EPR, and STM to accurately characterize the dynamic behavior of oxygen vacancies [127]. Calculate the oxygen vacancy formation energy and CO_2_ adsorption energy through DFT to provide theoretical guidance for material design [128]. In terms of scaling up, the high‐temperature reduction‐doping process in the laboratory needs to be converted into a continuous flow synthesis process [129]. In addition, during long‐term high‐temperature reactions, oxygen vacancies may be "filled" by the reaction atmosphere, so it is necessary to design an oxidation‐reduction cycling mechanism to achieve self‐recovery and maintain catalytic stability.

Therefore, oxygen vacancies serve as the core active sites for metal oxides and perovskites in the thermocatalytic conversion of CO_2_. Through methods such as doping, reduction, A/B site regulation, and interface engineering, their concentration and electronic properties can be precisely controlled, thereby significantly enhancing the activity, selectivity, and stability of CO_2_ hydrogenation or dry reforming [130]. By integrating emerging technologies such as photothermal and photocatalysis, it is anticipated that CO_2_ can be converted into high‐value resources at lower temperatures and with higher energy efficiency in the future.

## 5 Challenges

Despite significant progress in heterogeneous catalysis, its advancement toward large‐scale industrial application still faces several formidable challenges. A critical perspective in addressing these challenges is to recognize that they manifest and are prioritized differently along the two primary CO_2_ conversion pathways: thermocatalysis and electrocatalysis [131]. The following sections will analyze the core challenges of deactivation, selectivity, cost, and lifespan through this pathway‐dependent lens [132].

### 5.1 Inactivation Mechanism

The deactivation mechanisms summarized above in Table 3 are universal concerns in catalysis; however, their prevalence, driving forces, and manifestation details are strongly dependent on the specific CO_2_ conversion pathway.

**TABLE 3:** Deactivation mechanisms and stabilization strategies for CO_2_ conversion catalysts.

Inactivation type	Main manifestations of the inactivation type	Recent research progress	Literature
Sintering	Under high temperatures, metal particles agglomerate, and the specific surface area decreases, resulting in a sharp reduction in the number of active sites.	The formation of ultrafine metal atoms or sub‐nanoclusters on carriers through atomic layer deposition (ALD) can significantly inhibit sintering; related work has been systematically reviewed in several articles from 2024 to 2025.	[133, 134]
Carbon deposit	Intermediate polymerization forms a carbonaceous coating, blocking active sites.	Using core–shell structures or surface functionalization (such as introducing oxygen‐containing groups) can enhance the oxidation desorption rate of carbon. In Cu‐based systems, carbon deposition is suppressed through defect engineering.	[135]
Contamination (impurities such as sulfur, chlorine, etc.)	Trace impurities form strong coordination with the metal center, leading to deactivation of the active site.	By constructing a selective adsorption layer (such as a metal oxide thin film) on the surface of the carrier, sulfides can be captured and transformed, reducing the risk of poisoning.	[136]

In thermocatalytic processes (e.g., high‐temperature CO_2_ hydrogenation or dry reforming), sintering is a primary challenge due to the elevated operational temperatures (often > 300°C) that promote metal atom migration [137]. Carbon deposition (coking) is also a major issue, resulting from the undesired decomposition or deep dehydrogenation of carbon‐containing intermediates (e.g., CH_*x*_) on the catalyst surface under reducing, high‐temperature conditions [138]. Poisoning by impurities like sulfur (common in industrial flue gas or syngas) is a critical concern for long‐term stability [139].

In electrocatalytic CO_2_ reduction (ECO_2_R), the dominant stability challenges often differ [140]. While sintering can occur at high current densities due to localized heating or potential‐induced reconstruction, it is not as pervasive as in high‐temperature thermocatalysis. Severe carbon deposition is less common in aqueous ECO_2_R under mild conditions [141]. The more prevalent failure modes include: (i) Chemical/structural reconstruction of the catalyst surface (e.g., reduction of oxides, agglomeration, or phase change) driven by the applied potential and reaction environment [142]; (ii) Catalyst detachment or flooding in gas diffusion electrodes [143]; (iii) Degradation of ionomer or membrane components in membrane electrode assemblies (MEAs) [144]. Poisoning from feed gas impurities remains relevant but may involve different species (e.g., metal ions from the electrolyte or anode) [139].

Therefore, while the fundamental deactivation categories (sintering, coking, poisoning) provide a useful framework, the design of stabilization strategies must be tailored to the specific conditions and dominant degradation pathways of the targeted conversion technology (thermal vs. electrochemical).

### 5.2 Selective Regulation

The challenge of product selectivity is paramount for both pathways, but the underlying control parameters and design strategies diverge significantly. CO_2_ reduction can produce a variety of products, including C_1_ (CO, CH_4_, methanol) and C_2_^+^ (ethylene, alcohols), with complex electron‐proton transfer steps [145]. By precisely tuning the electronic structure of the metal center through the coordination environment, high selectivity for specific products can be achieved. For example, rare‐earth single‐atom catalysts show significant enhancement for C_2_^+^ products in CO_2_RR [146]. Coating a metal core with an oxide or sulfide shell can regulate the local electric field and adsorption energy, promoting multi‐step electron transfer processes [147]. Introducing functional groups such as amino and carboxyl groups on the metal surface to create a synergistic microenvironment, enhancing the selectivity for C_2_^+^ products [148].

### 5.3 Cost and Lifespan

Traditional, highly active catalysts are mostly based on precious metals, such as Pt, Pd, and Au, which are costly and have limited availability [149]. First‐row transition metals (Fe, Co, Ni) can be prepared through coordination polymerization or MOF precursors, significantly reducing costs while maintaining activity [150]. Non‐metallic catalysts (such as carbon‐based or nitrogen‐doped graphene) can also achieve high selectivity under specific conditions and possess good corrosion resistance [151]. Structural reconstruction under high temperatures and high current densities is the main obstacle [152]. The latest in situ/real‐time characterization techniques (such as operando X‐ray absorption and environmental TEM), combined with DFT kinetic simulations, have already been able to track atomic migration and phase transitions of catalysts under working conditions, providing a theoretical basis for designing reconstruction‐resistant systems.

### 5.4 Frontier Research Directions

The frontier research directions outlined above in Table 4 are critical for advancing all CO_2_ conversion technologies; however, their specific implementation, priority, and targeted outcomes are intrinsically shaped by the underlying conversion pathway (thermocatalytic vs. electrocatalytic) [155].

**TABLE 4:** Frontier research directions and key technologies for CO_2_ conversion catalysts.

Direction	Key technology/Method	Representative achievements	Reference
Advanced synthesis	Atomic layer deposition, cryogenic synthesis, and rapid thermal shock	Achieving uniform dispersion of sub‐nanometer metal atoms, significantly inhibiting sintering	[133]
Surface modification	Core‐shell structure, molecular layer self‐assembly, and covalent bonding of functional groups	Enhance the stability and selectivity of active sites	[153, 154]
In situ characterization theory	Operando XAS, environmental TEM, machine learning‐assisted DFT	Revealing the structural evolution and deactivation pathways of catalysts in CO_2_RR	[135, 136]
Molecular/metal synergy	Immobilize highly selective molecular catalysts onto solid supports to achieve the dual advantages of "molecular" and "heterogeneous" catalysis.	Achieving single‐molecule level activity and selectivity enhancement in electrochemical CO_2_RR	[153]
Sustainable materials	Preparation of high‐porosity carriers using renewable carbon sources (biomass charcoal, MOF‐derived carbon)	Reduce overall costs while improving electrical conductivity and mass transfer.	[154]

For the thermocatalytic pathway, the emphasis of these directions is adapted to high‐temperature and high‐pressure environments [156]. Advanced synthesis (e.g., ALD) focuses on creating nanostructures resistant to sintering and coke formation [157]. Surface modification aims to engineer interfaces (e.g., core–shell) that enhance thermal stability and suppress undesirable side reactions like methane formation in methanol synthesis [158]. In situ characterization is pushed to operate under realistic high‐temperature/pressure conditions to decipher the dynamic state of active sites and reaction intermediates [159]. Molecular/metal synergy in this context often involves grafting molecular complexes onto robust supports to combine molecular precision with heterogeneous stability for gas‐phase reactions [5]. Sustainable materials research prioritizes the development of cost‐effective, thermally stable carriers from abundant resources [160].

For the electrocatalytic pathway (ECO2R), the focus shifts to the electrochemical interface [140]. Here, advanced synthesis and surface modification are geared toward creating nanostructures that maintain activity and selectivity under applied potential and in aqueous electrolytes, with a strong emphasis on controlling crystal facets and defects to steer product distribution [161]. In situ characterization is paramount for capturing potential‐dependent surface reconstruction and the behavior of key intermediates (e.g., *CO) in real‐time [162]. Molecular/metal synergy is a highly active frontier, as seen in the table, precisely aiming to heterogenize molecular catalysts for ECO_2_R to achieve unparalleled selectivity [163]. Sustainable materials development is crucial for fabricating conductive, porous electrodes and replacing expensive components (e.g., Nafion) in electrolyzers.

Therefore, a clear pathway‐specific lens is essential when evaluating and pursuing these frontier directions [164]. Future breakthroughs will depend not only on advancing these technologies generically but also on tailoring them to address the distinct scientific and engineering challenges posed by either thermal or electrical driving forces [165].

### 5.5 Prospects and Recommendations

To promote the practical application of CO_2_ catalytic conversion technology, the following directions are crucial. First, it is necessary to adopt a multi‐scale collaborative design strategy, synchronously designing from the atomic scale, where single atoms and defect regulation are considered, to the macroscopic scale, where pore size and morphology optimization are considered, in order to construct highly efficient catalysts with a hierarchical network of active sites [166]. Second, efforts should be focused on full‐process system integration, coupling the CO_2_ capture, conversion, and product separation stages into an integrated process, thereby significantly reducing system energy consumption and enhancing the economic feasibility of the entire technical route [167]. In terms of catalyst evaluation, it is essential to establish standardized protocols for deactivation testing. By uniformly assessing deactivation pathways such as sintering, coking, and poisoning, effective comparison and benchmarking of different research data can be achieved. In addition, data‐driven methods such as machine learning should be fully utilized to extract features and train models from massive amounts of literature and experimental data, enabling the rapid and accurate prediction of the activity and lifespan of new catalytic materials. This approach significantly accelerates the development of high‐performance catalysts. Through such multi‐faceted collaborative innovation, it is expected to break through the current bottlenecks in CO_2_ utilization.

Significant progress has been made in multiphase catalysis for the large‐scale utilization of CO_2_, but it still faces four major challenges: deactivation, selectivity, cost, and lifespan. By combining advanced synthesis methods, such as atomic layer deposition, core–shell structures, and surface functionalization, with in‐depth insights from in situ characterization and theoretical calculations, it is possible to precisely regulate active sites at the microscopic level, thereby enhancing the selectivity of multi‐carbon products and prolonging the catalyst's lifespan [39, 106]. Future research needs to advance collaboratively in the areas of material innovation, process integration, and intelligent screening in order to achieve truly industrial‐scale applications.

## 6 Homogeneous Catalysis

The following chapter focuses on the application of homogeneous molecular catalysts and conceptually related single‐atom catalysts within the context of electrocatalytic and, to a lesser extent, photocatalytic CO_2_ reduction [168]. The discussion centers on systems where catalytic transformations are driven by electrical or photonic energy input, often in liquid electrolytes [169]. This distinguishes the core content of this chapter from the design and application of molecular catalysts in thermal hydrogenation reactions, which operate under distinct principles (e.g., CO_2_ insertion into metal‐hydride bonds) and are more relevant to the thermocatalytic pathway discussed in earlier sections. The performance metrics, mechanistic studies, and design strategies highlighted herein—such as Faradaic efficiency, overpotential, and proton‐coupled electron transfer management—are primarily pertinent to the electrochemical (and photoelectrochemical) conversion pathway [56].

The core advantage of homogeneous CO_2_ reduction catalysis lies in the well‐defined structure of the active center and the precisely tunable ligand environment, enabling atomic‐level control over reaction pathways and product selectivity [170]. The electronic effects and steric hindrance of ligands, as well as the secondary coordination sphere (such as proton donors or hydrogen bond acceptors), can regulate the activation of CO_2_ and the formation of *COOH intermediates at the molecular level, enabling the catalyst to achieve highly selective conversion at low overpotentials [171]. Taking Re(I) complexes carrying local proton sources as an example, studies have shown that they can reduce CO_2_ to formate under electrochemical and photochemical conditions, with product selectivity exceeding 74% and a turnover number (TON) greater than 80 [172, 173]. Further mechanistic studies using infrared spectroelectrochemistry and DFT calculations revealed the key role of ligand–metal cooperation in lowering the reduction potential and suppressing hydrogen evolution (HER) [174]. Similarly, Re(I)‐NHC complexes achieved a photovoltaic voltage of over 440 mV on silicon nanowire photoelectrodes, significantly enhancing the selectivity for CO and demonstrating the potential of coupling metal complexes with semiconductor photoelectronic materials [175].

In recent years, single‐atom catalysts (SACs) have offered structural uniformity similar to that of homogeneous systems, while also exhibiting high atomic efficiency [176]. The dispersion of Ni single atoms in graphene vacancies can achieve over 95% CO selectivity at a 550 mV overpotential and remain stable during 20 h of continuous electrolysis, indicating that single‐atom sites can significantly suppress the HER and enhance CO_2_RR activity [177]. Ni single‐atom catalysts prepared via metal–organic framework (MOF) ion exchange exhibited a high turnover frequency (TOF) of 5273 h^−1^ and a Faradaic efficiency of over 71.9%, further confirming the decisive role of the ligand‐metal coordination environment in activity [178, 179]. In terms of coordination number regulation, low‐coordination Ni‐N_3_ sites (compared to Ni‐N_4_) significantly enhance the formation rate of the COOH intermediate, enabling a Faradaic efficiency for CO of 95.6% [180]. Ni single atoms supported on defect‐rich zirconia achieve 92.5% CO selectivity under visible light without sacrificial agents, indicating that single‐atom sites can suppress water splitting and promote CO_2_→CO conversion under photocatalytic conditions. In addition, embedding Ni single atoms into covalent organic frameworks (COFs) to form a Ni‐TpBpy catalyst produces 4057 µmol g^−1^ of CO within a 5‐hour reaction, with a selectivity as high as 96%, and maintains 76% selectivity even at low CO_2_ partial pressure (0.1 atm), further demonstrating the synergistic effect of pore structure on CO_2_ adsorption and activation. In more complex multi‐metal cooperative systems, a mixed catalyst of single‐atom Ni and nanoscale Cu achieves efficient CO_2_→CO production and promotes C—C bond coupling via CO accumulation on the Cu surface, ultimately reaching 66% Faradaic efficiency for ethylene (C_2_H_4_) in an alkaline flow cell, illustrating the pathway by which single atoms and nanoscale metals synergistically enhance multi‐carbon product formation [181].

Overall, homogeneous catalysis enables the visualization and tunability of active centers through molecular design, making it possible to conduct in‐depth studies of the CO_2_ reduction mechanism [52]. Single‐atom catalysis, on the other hand, provides a nearly homogeneous electronic structure while maintaining structural uniformity. The combination of the two offers a multidimensional design space for achieving highly selective and low‐energy CO_2_ conversion [176]. Future research directions can focus on (1) functionalizing the secondary environment of ligands to reduce the formation energy barrier of COOH further [182]; (2) Combine single‐atom sites with interface engineering of photosensitive materials or electrolytes to achieve photoelectrochemical synergistic driving [183]; (3) Achieving spatial separation and high‐density distribution of active sites through ordered supports such as MOFs/COFs, thereby enhancing the scalability of practical processes while maintaining the advantages of homogeneous systems [184]. Such systematic regulation at the molecular and atomic levels will lay a solid foundation for the industrialization and diversification of CO_2_ reduction products.

### 6.1 Molecular Catalyst

Homogeneous molecular catalysts are usually centered on organometallic complexes with well‐defined molecular structures (such as bipyridine‐metal complexes, phosphine‐metal complexes) or macrocyclic compounds (such as metal porphyrins, metal phthalocyanines). Their catalytic performance highly depends on the synergistic effect between the central metal ion and the ligand environment [185].

#### 6.1.1 The Decisive Role of Central Metal Ions in Catalytic Pathways

Cobalt (Co): Co‐porphyrin exhibits very high selectivity in the electroreduction of CO_2_ to CO, with a Faradaic efficiency of over 90% and a moderate overpotential [186].In the photocatalytic system, Co‐phthalocyanine (CoPc) facilitates the multi‐electron conversion of CO_2_ to methanol, with the key step being the protonation of *CHO. Alkali metal ions (Li^+^ > Na^+^ > K^+^ > Cs^+^) can accelerate proton‐electron coupling and enhance the yield [187].

##### 6.1.1.1 Rhenium (Re)

Re‐bipyridine tricarbonyl complexes (fac‐[Re(bpy)(CO)_3_X]^n+^) can directly capture CO_2_ in photo/electro dual‐mode catalysis, forming a detectable reduced tetracarbonyl intermediate [Re(bpy)(CO)_4_]^0^, which then releases CO, providing a complete reaction pathway. The electronic properties of the ligands significantly affect the stability of the intermediate, and electron‐withdrawing groups in the ligands can increase the CO_2_ binding energy, thereby enhancing the catalytic activity [188].

##### 6.1.1.2 Palladium (Pd)

A Pd monolayer on Pt(111) (PdML/Pt) achieves high selectivity for CO_2_ to formic acid (HCOOH) (≈95% FE) at low overpotentials, while CO poisoning occurs at more negative potentials, indicating that Pd has weak adsorption for the *COOH intermediate, which helps suppress CO formation. The Pd–Bi alloy further inhibits HER and CO production, maintaining about 95% HCOOH selectivity even at a current density of 200 mA  cm^−2^.

##### 6.1.1.3 Tin (Sn)

Sn‐based single‐atom or nanoscale catalysts can reduce CO_2_ to HCOOH in acidic electrolytes at nearly zero overpotential, with Faradaic efficiencies often exceeding 90% and minimal competition from the hydrogen evolution reaction (HER). Ligands or microstructures on supports (such as N‐ and S‐co‐doped graphene frameworks) can further increase the density of active sites, resulting in a production rate of 5 mol h^−1 ^g^−1^ [189].

#### 6.1.2 Key Strategies of Ligand Engineering

The ligand engineering strategies summarized above in Table 5 are fundamental tools for tuning molecular catalyst performance. However, their application and primary objectives are distinctly shaped by the catalytic pathway. In thermocatalytic systems (e.g., CO_2_ hydrogenation in organic solvents or under thermal conditions), ligand design often aims to facilitate CO_2_ insertion into metal‐hydride bonds, stabilize alkyl/alkoxide intermediates, and control the selectivity between oxygenates (like formate or methanol) and hydrocarbons. In contrast, for electrocatalytic CO_2_ reduction, the focus of ligand engineering shifts dramatically towards modulating the metal center's redox potential, optimizing the binding strength of key electrochemical intermediates (like COOH or CO), and precisely managing proton delivery to suppress the competing hydrogen evolution reaction (HER)—as exemplified by most cases in the table. Even within electrocatalysis, strategies may differ between purely electrochemical and photoelectrochemical driver systems. Therefore, while the categories of strategies (electronic, steric, etc.) are universal, their implementation must be precisely tailored to the specific reaction milieu (thermal vs. electrochemical) and the desired product profile.

**TABLE 5:** Ligand engineering strategies for molecular Co_2_ conversion catalysts.

Strategy	Mechanism of action	Representative example	Literature
Electronic effects (electron‐donating/electron‐withdrawing groups)	Adjust the electron density of the metal center to alter the adsorption energies of intermediates, such as *CO_2_^‐^, *COOH, and *OCHO.	In the Re‐bpy system, introducing electron‐withdrawing groups significantly enhances CO_2_ binding and reduces overpotential; in Co‐porphyrin, adding hydroxyl groups (—OH) forms a hydrogen bond network, lowering the energy barrier of the CO_2_^‐^ intermediate.	[190, 191]
Steric hindrance	Bulky ligands prevent excessive *H^+^/*H adsorption, suppress hydrogen evolution, and guide C—C coupling.	Introducing quaternary ammonium groups into Fe‐porphyrin generates a local electric field, enhancing the stability of *CO_2_^‐^ and suppressing HER. Bulky ligands can prevent *CO formation, thereby improving the selectivity of CO_2_→CO.	[192]
Positive charge / quaternary ammonium group	By electrostatically attracting CO_2_^‐^, the local CO_2_ concentration is increased and proton transfer is promoted.	The Fe porphyrin quaternary ammonium group achieves a localized electric field effect on *CO_2_^‐^, significantly reducing the overpotential and enhancing the rate.	[192]
Secondary coordination sphere (hydrogen bonding, π‐π interactions)	Through non‐covalent interactions between the ligand side chain and the intermediate, additional stabilization or activation pathways are provided.	Using a hydroxy–hydroxy hydrogen bonding network, o‐Cu‐Por‐Sa achieves an 84% CH_4_ FE in CO_2_RR, demonstrating the dual control of ligand conformation on selectivity; the second coordination sphere of organocobalt complexes enables CO_2_ insertion into metal hydrogen bonds or direct hydrogenation in photocatalysis.	[191, 193]
Fixation method / Support effect	Anchor molecular catalysts onto conductive supports through non‐covalent, covalent, or coordination bonds to regulate electronic coupling and the exposure of active sites.	The effect of support‐regulated Cu pore aperture on *CO coverage led to an increase in C_2_^+^ yield from 14% to 65%; different immobilization modes (non‐covalent, covalent, coordination) result in significant differences in the product distribution of Cu porphyrin CO_2_RR.	[194]

#### 6.1.3 Recent Research Hotspots and Achievements

Recently, research on the electrochemical reduction of carbon dioxide has achieved several important breakthroughs in achieving high selectivity for specific products. In terms of high selectivity for CO production, molecular catalysts have performed exceptionally well. For example, Co‐porphyrin and Re‐bpy catalysts, which are tuned by ligand electronics, have achieved Faradaic efficiencies for CO production exceeding 90% and 80%, respectively [195].

Beyond CO, progress has also been made in the selective synthesis of formic acid and methanol. The Pd–Bi alloy catalyst can still maintain a selectivity of 95% for formic acid at an industrial‐level current density of 200 mA cm^−2^ [196]. The Co‐Pc catalyst successfully reduces CO_2_ to methanol in an alkaline electrolyte, and the protonation process of the key intermediate *CHO was found to be effectively regulated by the ionic shell at the electrode interface [197].

**FIGURE 8:**
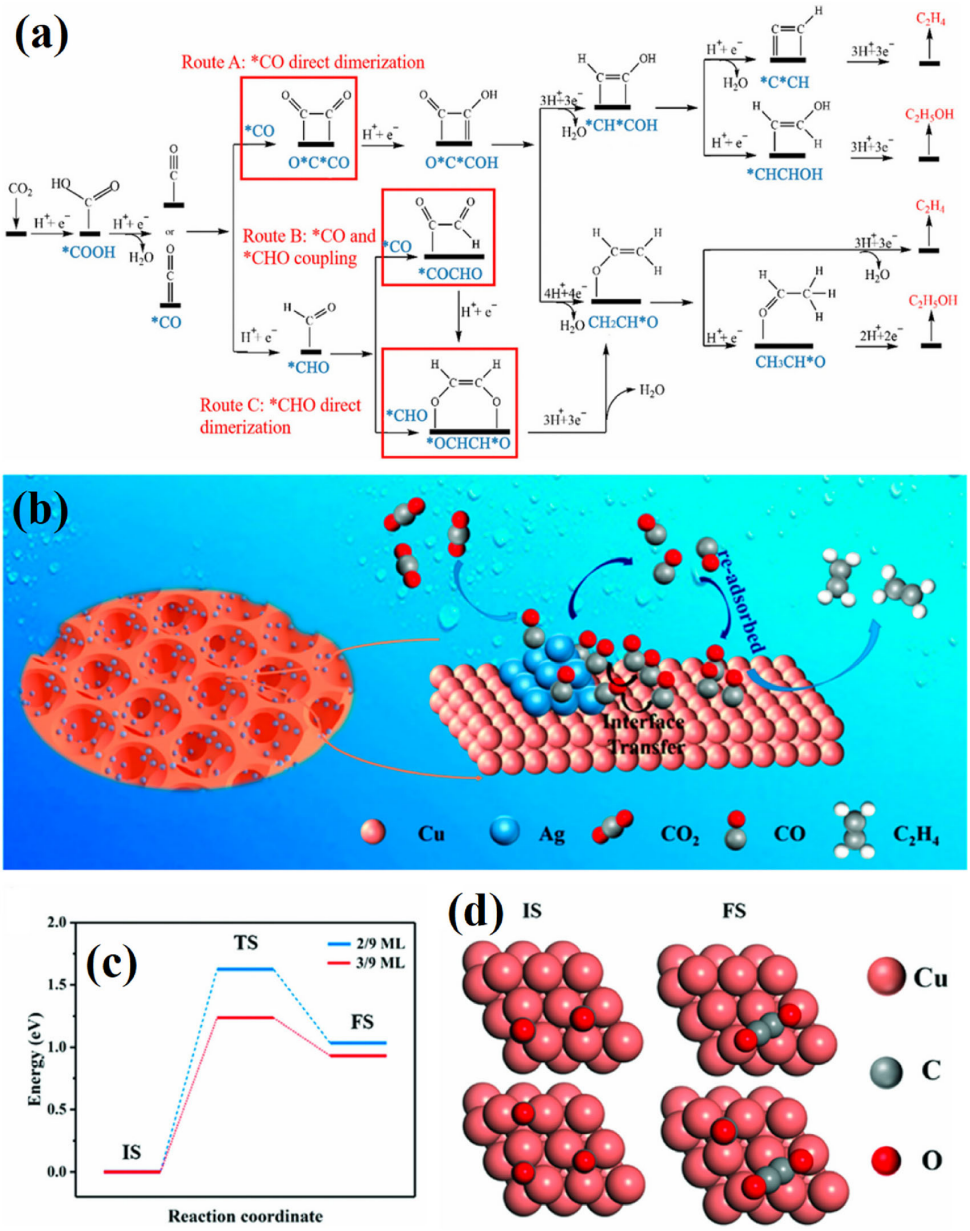
(a) Various reaction routes in producing alcohol and ethylene through the CO_2_RR process [74]. (b) The mechanism of ethylene generation onto Cu/Cu_2_O‐Ag through CO_2_RR [74]; (c) activation energy barriers [74]; and (d) initial state (IS) and final state (FS) configurations on the coverage of CO [74]. Reproduced with permission. Adapted from [74]. Copyright 2025, MDPI.

In the field of synthesizing higher‐value multi‐carbon products (C_2_^+^, C_3_^+^), research hotspots focus on enhancing C—C coupling through catalyst design (Figure 8a–d) [74]. As shown in Figure 8a, CO_2_ reduction can branch via multiple pathways such as *CO—*CO and *CO—CHO to produce ethanol and ethylene. Figure 8b–d further takes the Cu/Cu_2_O‐Ag electrode as an example, revealing that a monolayer (ML) of Ag modification on the Cu foam surface can regulate CO coverage and lower the C—C coupling activation barrier (˜0.48 eV). Its IS/FS configuration shows that the CO—*CO intermediate at the Cu–Ag interface has a shortened distance and enhanced bonding, thereby significantly improving ethylene selectivity, providing an expandable design paradigm for "interface engineering → CO coverage → C_2_^+^ products" [74]. For example, the Cu/CoPc tandem catalyst achieves about 52% Faradaic efficiency for C_2_^+^ products (ethylene) by providing a large amount of *CO intermediates to the Cu sites [198]. The Mo_1_Cu single‐atom alloy further increased the Faradaic efficiency of C_2_^+^ products to about 86% at a high current density of 0.8 A cm^−2^. Particularly noteworthy is that the bimetallic Cu–binuclear complex achieved, for the first time, the molecular catalytic synthesis of C_3_ products (n‐propanol) under room temperature light irradiation, thereby opening a new path in this field [199].

In addition, for practical applications, the direct utilization of low‐concentration CO_2_ has become an emerging frontier. Research has shown that using Rh‐bpy‐Cp* molecular catalysts in combination with electrodes modified with positively charged imidazolium layers can selectively produce formic acid in environments simulating industrial waste gas (5%–10% CO_2_), fully demonstrating the great potential of the synergistic strategy of molecular catalysis and electrode interface modification [200].

### 6.2 Solvent Effect

In a homogeneous catalytic system, the solvent is far more than just a reaction medium; it directly participates in the reaction and has a profound impact on catalytic performance.

#### 6.2.1 Solubility and Mass Transfer: Solvent Structure Determines CO_2_ Interfacial Concentration

The microscopic structure of the solvent is a key factor in determining CO_2_ solubility and mass transfer efficiency [201]. For ionic liquids (ILs), the polarity index and free volume of their cations and anions are key parameters affecting the volumetric solubility of CO_2_ [23]. Generally speaking, the greater the polarity and the higher the free volume, the better the solubility of CO_2_, and the lower the Henry's constant. Among these, the type of anion plays an important role; it has been traditionally believed that fluorinated anions (such as [Tf_2_N]^‐^) can enhance solubility, but studies have found that certain cyano anions (such as [N(CN)_2_]^‐^) perform even better. Even in multivalent ILs, the effect of the anion can surpass the dominant role of free volume in regions of low free volume [71].

In addition, mass transfer processes can be effectively enhanced through solvent engineering. For example, by immobilizing ionic liquids (ILs) on porous supports to form supported ionic liquid membranes, the interfacial effects and increased free volume can enhance CO_2_ solubility by 1.2–3.9 times [202]. In supercritical CO_2_ systems, the introduction of co‐solvents such as ethanol and DMSO can also significantly enhance the solubility of Ils [203]. In an aqueous system, the pH value regulates the solubility of CO_2_ by affecting the equilibrium of its conversion to carbonic acid, with an acidic environment generally being more favorable [204].In a water‐based system, the pH level controls the solubility of CO_2_ by influencing the balance of its transformation into carbonic acid, with acidic conditions typically enhancing this solubility [205].

#### 6.2.2 Proton Source and Reaction Pathways: Precisely Controlling Product Distribution

The characteristics of the proton source and its delivery mechanism are another decisive factor in determining the selectivity of CO_2_ reduction products.

First, the strength of the proton source (pKa) directly influences the reaction pathway. In electrocatalytic systems, using a strong proton source with a lower pKa (such as methanol or hydrofluoric acid) can accelerate the initial protonation of CO_2_. Still, it often exacerbates the hydrogen evolution side reaction, leading to a decrease in the selectivity of the desired product (such as CO) [206, 207]. Therefore, by finely tuning the acidity of the proton source, it is possible to achieve a competitive balance between CO_2_ reduction and hydrogen evolution reactions, thereby switching the main product [208].

Secondly, the spatial position and delivery method of protons are crucial. Conceptually, a "proton fence" formed by *OH adsorbed on sulfur vacancies can use spatial hindrance to restrict multi‐proton transfer, thereby locking the product as CO or CH_4_ [209]. At the molecular level, designing built‐in proton donors is an efficient strategy. For example, in Mn‐bpy‐R or iron‐porphyrin molecular catalysts, adjacent hydroxyl groups (—OH) can act not only as proton donors in non‐protic solvents but also as hydrogen bond acceptors. Adding a trace amount of weak acid can increase the CO_2_ reduction rate by nearly one hundred times and switch the product from CO to formic acid [210]. This reveals the significant impact of precisely constructing the local proton environment on activity and selectivity.

Ultimately, the solvent and electrolyte environments jointly shape the proton‐coupled electron transfer pathway [154]. In an alkaline electrolyte, the concentration of H_3_O^+^ is suppressed, and the product on the silver electrode shifts from pure CO to formic acid, demonstrating that protons are the key intermediates [211]. In non‐aqueous organic solvents, the reaction is highly dependent on the addition of trace water or a weak acid as a proton source; otherwise, the conversion rate will be extremely limited [212]. The reaction pathways on metal surfaces are also greatly influenced by this. For example, the Pb(111) surface, under different solvent and cation environments, can switch the product from HCOOH to CO by stabilizing different intermediates [197]. In Cu‐based catalysts, adjacent nitrogen atoms can act as proton relay stations, promoting C—C coupling and thereby achieving high selectivity for C_2_^+^ products [148].

#### 6.2.3 Comprehensive Insights and Design Strategies

Therefore, the solvent system collaboratively regulates CO_2_ catalytic conversion through multiple mechanisms:

##### 6.2.3.1 Physical Dissolution

The polarity of the solvent, free volume, and anion structure collectively determine the concentration of CO_2_ in the liquid phase, providing sufficient interfacial reactants for the reaction [213].

##### 6.2.3.2 Proton Management

The system's pH value, trace water, and the strength of added proton sources together constitute the proton supply network, whose concentration, spatial distribution, and transport efficiency directly determine the reaction pathway and product selectivity [214].

##### 6.2.3.3 Pathway Switching

By carefully designing solvent combinations (such as IL/water/organic solvent mixtures) and matching suitable proton donors, it is entirely possible to achieve the selective regulation of products from CO, HCOOH, CH_4_, to C_2_^+^ chemicals on the same catalytic platform [215].

Therefore, the design of future catalysts and reaction systems must treat the solvent as an active and functional component, achieving precise control over the high‐value conversion of CO_2_ through cross‐scale synergistic optimization.

### 6.3 Limitations of Homogeneous Catalysis

Homogeneous catalysts exhibit extremely high activity and selectivity in the laboratory; however, their large‐scale industrial application is constrained by both the difficulty of recovery and insufficient stability. Traditional separation methods, such as distillation and extraction, are costly and prone to loss of precious metals, especially since organometallic complexes sensitive to air and water can easily undergo ligand dissociation or decomposition under long‐term or high‐reduction‐potential electrocatalytic conditions, leading to deactivation [216]. To overcome these bottlenecks, current research focuses on two main directions: reinforcing ligand structures and immobilizing or hybridizing catalysts.

At the ligand level, rigid polydentate ligands (such as carbenes and U_2_‐NHC) significantly enhance the chemical and electrochemical tolerance of the metal center by providing stronger σ‐donation and steric hindrance, allowing it to remain active in air and resistant to dissociation at high potentials. Meanwhile, to address the issue of recovery, researchers anchor molecular catalysts onto solid supports via covalent bonding, ionic adsorption, or non‐covalent adsorption, thereby achieving the "heterogenization of homogeneous catalysts" [5]. Covalent immobilization has been successfully achieved on materials such as carbon nanotubes, silica, and γ‐Al_2_O_3_, retaining the high selectivity of molecular catalysts while providing the advantages of easy separation and reuse [217]. Ion adsorption utilizes electrostatic interactions between charged polymers (such as Nafion) or functionalized alumina surfaces to adsorb catalysts onto the carrier surface, enabling reversible loading and release, which is suitable for adjustable flow reaction systems [218]. In terms of non‐covalent adsorption, functionalized graphene oxide provides a stable support platform for molecular catalysts due to its large specific surface area and tunable electronic structure, exhibiting excellent activity and selectivity in organic transformations, such as oxidation and esterification [219].

**FIGURE 9:**
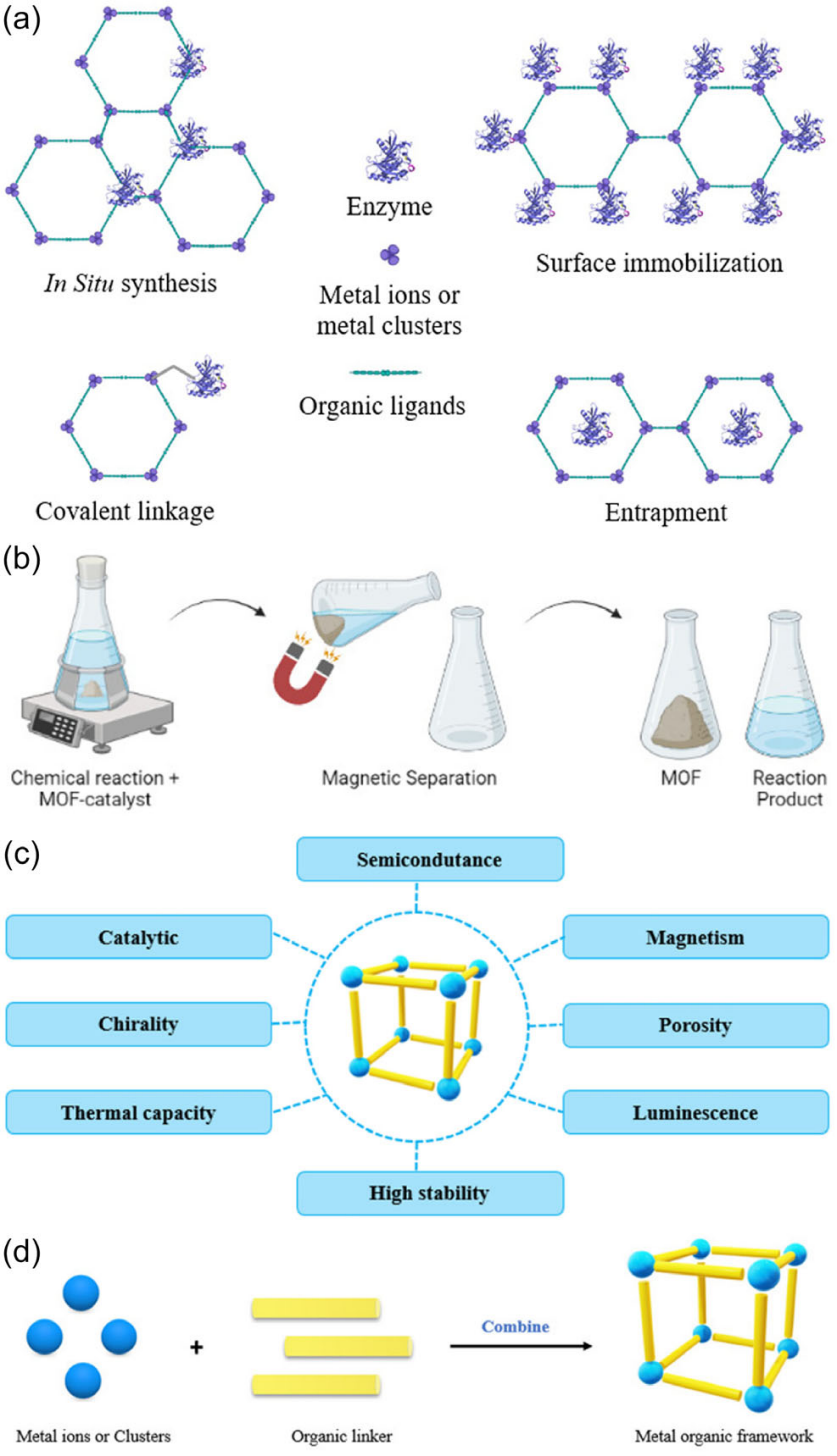
(a) Different techniques of enzyme immobilization onto MOFs [221]. (b) The separation of MOFs from their reaction media by their magnetic properties, which enables their simplified removal—an excellent characteristic for catalysts [221]. (c) Composite properties based on MOF structures, highlighting their thermal capacity, chirality, high stability, semiconductivity, luminescence, magnetism, catalytic power, and porosity [221]. (d) The formation of a crystalline structure of MOFs based on organic ligands being coupled to a metallic center [221]. Reproduced with permission. Adapted from [221]. Copyright 2022, MDPI.

In the further advancement of immobilization, metal‐organic frameworks (MOFs) are utilized as three‐dimensional porous networks to embed homogeneous catalysts into their lattices or ligand sites, thereby achieving true heterogeneous catalysis [184]. MOF‐based catalysts have demonstrated high activity, recyclability, and structural designability in various reactions, including plastic depolymerization, N‐heterocycle dehydrogenation, and enzyme mimicry, while maintaining their crystalline integrity even after multiple cycles of use [220]. These strategies together constitute the core framework of the current research on "heterogenization of homogeneous catalysts": enhancing the intrinsic stability of the metal center through rigid ligands; achieving efficient recovery and reuse through covalent, ionic, or non‐covalent immobilization (Figure 9a,b) [221]. Using MOFs and other porous crystals to provide a tunable microenvironment, balancing activity and selectivity (Figure 9c,d) [221]. Future research and development must continue to focus on computationally driven ligand design, precise regulation of carrier surface chemistry, and achieving a cost‐benefit balance in process scaling, in order to realize the true industrial implementation of homogeneous catalysts in energy, chemical, and fine synthesis fields.

## 7 Single‐Atom Catalyst

The following section discusses single‐atom catalysts (SACs) as a unique class of materials that demonstrate significant potential across bothmajor CO_2_ conversion pathways: thermocatalysis and electrocatalysis [168]. Their discussion here serves as a conceptual bridge between the two technological domains. It is crucial to recognize that while the fundamental advantages of SACs—maximum atom utilization and uniform, tunable sites—are universal, their specificperformance targets, active site design criteria, and stabilization strategies are deeply pathway‐dependent. For instance, a Ni‐N‐C SAC designed for high‐selectivity CO production in electrocatalysisoperates under a potential‐driven mechanism to stabilize the COOH intermediate, whereas a Pt_1_/CeO_2_ SAC for selective methanol synthesis in thermocatalysis functions via facilitating H_2_ heterolytic dissociation and specific CO_2_ activation [222]. This chapter will present applications and challenges for SACs in both contexts, with the understanding that the underlying scientific principles are tied to the respective conversion environments (electrochemical vs. thermal) as established in preceding sections [223].

Single‐atom catalysts have been a groundbreaking advancement in the field of catalytic materials science in recent years. By anchoring metal atoms in an isolated form on suitable carrier surfaces, they maximize the utilization efficiency of metal atoms, achieving a paradigm shift from "nano catalysis" to "atomic catalysis" [224]. The unique structure of SACs makes them an ideal bridge between homogeneous catalysis (with well‐defined active centers) and heterogeneous catalysis (stable and easily separable), demonstrating great potential in CO_2_ catalytic conversion.

### 7.1 Unique Characteristics of Single‐Atom Catalysts

The core feature of SACs is that their active sites are composed of dispersed, isolated metal atoms, which are usually stabilized by coordination with heteroatoms (such as N, O, S) on the surface of the support. This structure brings numerous extraordinary properties

#### 7.1.1 Maximum Atomic Utilization Efficiency

Nearly 100% of metal atoms are exposed as active sites, eliminating the wastage of atoms "hidden" in the bulk of traditional nanoparticles, which is particularly economically valuable for catalysts rich in precious metals [225].

#### 7.1.2 Uniform and Tunable Active Sites

Since the active centers are isolated metal atoms, their coordination environment (M‐X_*n*_, such as M‐N_4_ or M‐O_4_) is highly uniform, providing a foundation for achieving highly selective catalysis [226]. By changing the type of support, doping elements (such as B, N, P, S), or the coordination number, the electronic structure of the metal center can be precisely controlled at the atomic scale, thereby optimizing its adsorption energy for CO_2_ and its reaction intermediates (such as *COOH, *CO) and guiding the reaction pathway [227].

#### 7.1.3 Unique Reaction Mechanism

**FIGURE 10:**
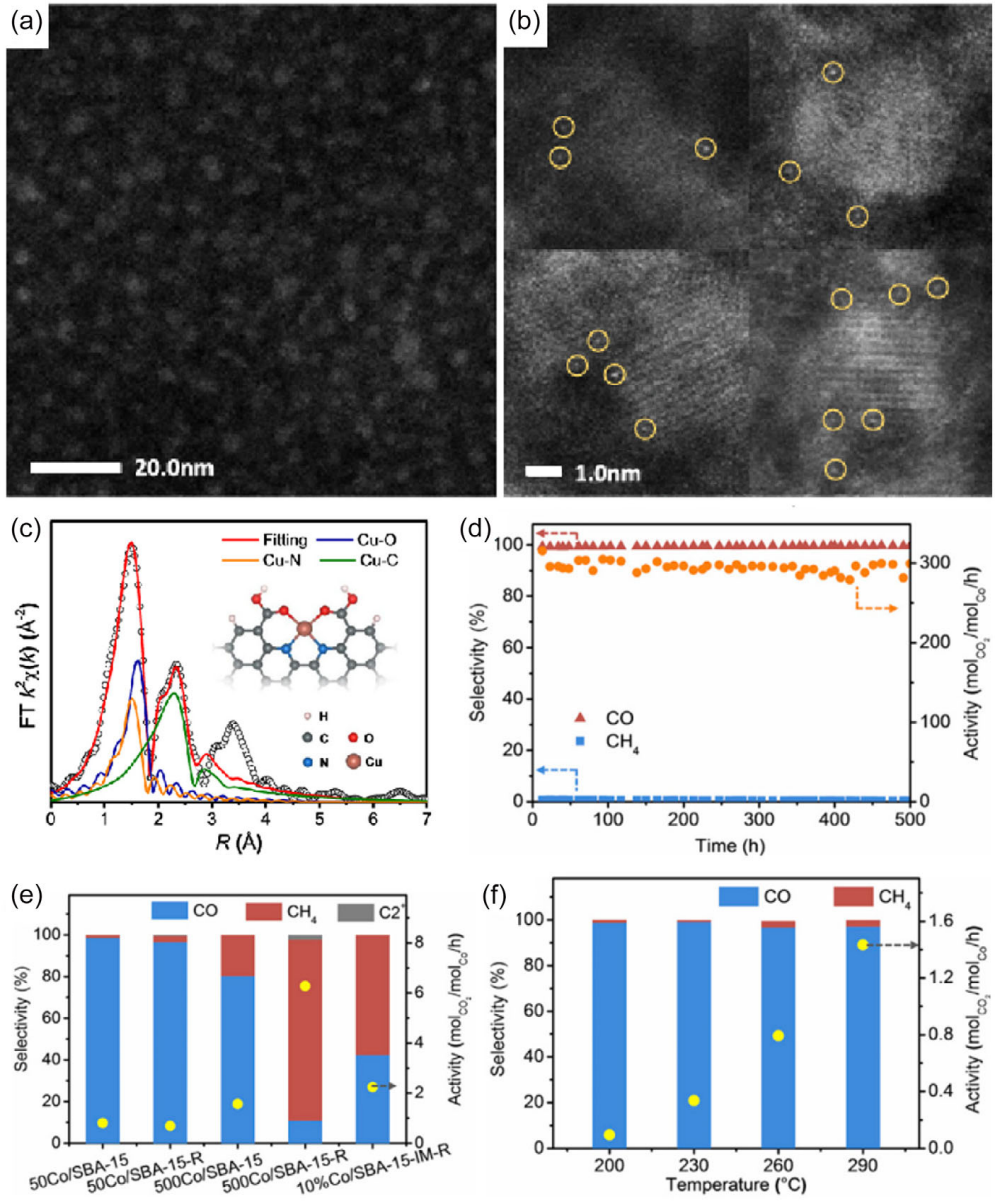
(a) Relatively large field of view and (b) typical view of HAADF–STEM images of distributed single Cu atoms in carbon dots [228]. Yellow circles in (b) indicate typical single Cu atoms [228]. (c) EXAFS fitting curves of Cu‐CDs in R space using backscattering paths of Cu–N, Cu–O, and Cu–C [228]. Reproduced with permission. Adapted from [228]. Copyright 2021, Springer Nature. (d) Stability of 50Co/SBA‐15 at 600°C and 0.1 MPa for 500 h [229]. (e) Catalytic performance of nCo/SBA‐15 and nCo/SBA‐15‐R at 260°C and 2 MPa [229]. (f) Catalytic performance of 50Co/SBA‐15 at 200°C–290°C and 2 MPa [229]. Reproduced with permission. Adapted from [229]. Copyright 2022, Elsevier.

Isolated single‐atom sites are usually unfavorable for reactions that require adjacent sites (such as C—C coupling), which gives them an intrinsic high selectivity for CO_2_ reduction to a single product (such as CO) (Figure 10a–c) [228, 230]. However, recent studies have shown that by designing dual‐atom sites or regulating the local reaction microenvironment, it is also possible to drive the generation of multi‐carbon products, which broadens the application scope of SACs [196].

### 7.2 Applications of Single‐Atom Catalysts

In CO_2_ electrocatalytic reduction, SACs have become a class of star materials. Among them, the most extensively studied SACs are those in which transition metals are loaded on nitrogen‐doped carbon supports (M‐N‐C, M = Ni, Fe, Co, Mn, etc.).

#### 7.2.1 Highly Selective CO Production

Ni—N—C and Fe—N—C SACs can reduce CO_2_ to CO with high selectivity, with Faradaic efficiencies typically exceeding 90% and even approaching 100% [231]. Its active sites are generally considered to be metal‐N_4_ coordination structures embedded in a carbon matrix. For example, the Ni—N_4_ site exhibits a moderate adsorption strength for the *COOH intermediate, resulting in a low overpotential and high turnover frequency [232].

#### 7.2.2 Exploration of Multi‐Carbon Product Formation

Despite significant challenges, initial successes in converting CO_2_ to multi‐carbon products such as ethylene and ethanol have been achieved by constructing adjacent metal atom pairs (such as Cu—Cu dimers or heteronuclear bimetallic sites like Cu—Zn), or by using functional groups on support surfaces to create a local environment enriched in *CO, demonstrating the potential of SACs in complex reactions.

#### 7.2.3 Thermal Catalytic Applications

In the CO_2_ hydrogenation reaction, SACs also exhibit excellent performance (Figure 10d–f) [229]. For example, Pt_1_ and Pd_1_ single‐atom catalysts supported on CeO_2_ or ZnO exhibit much higher selectivity than their corresponding nanoparticles in CO_2_ hydrogenation to methanol or reverse water‐gas shift reactions, which is attributed to the heterolytic dissociation of H_2_ at single‐atom sites and the specific activation of CO_2_ [233].

### 7.3 Challenges and Future Directions

Although single‐atom catalysts (SACs) show great potential in enhancing catalytic activity and selectivity, the transition from fundamental research to practical applications still faces significant challenges. First, controllable synthesis and stability are key bottlenecks. Current synthesis methods, such as wet impregnation and high‐temperature pyrolysis, struggle to ensure that all metal atoms exist in isolated forms and have limited yield; under real reaction conditions, such as high potentials or high temperatures, metal single atoms tend to migrate and aggregate due to their high surface energy, leading to deactivation [234]. Therefore, developing atomic‐level deposition techniques such as atomic layer deposition and electrochemical displacement, and designing robust supports with strong metal–support interactions (such as defect metal oxides and metal‐nitrogen coordination sites) have become research focuses. Secondly, precisely correlating structure with performance remains challenging. In situ identification of active sites requires the combination of advanced characterization techniques such as X‐ray absorption fine structure (XAFS) and scanning transmission electron microscopy (STEM), capturing their dynamic evolution under reaction conditions in real time; meanwhile, the introduction of multi‐scale computations and machine learning models helps establish a true “structure‐performance” relationship [235]. Future research will focus on three areas: first, designing novel carriers, such as porous polymers, covalent organic frameworks, and metal oxides with enhanced anchoring abilities through defect engineering, to improve the dispersion and thermal stability of single atoms [22]. Second, develop diatomic or atomic cluster catalysts to utilize the synergistic effects between adjacent metal atoms for multi‐step complex reactions, which show particular advantages in the synthesis of multi‐carbon CO_2_ products [181]. Third, it is necessary to explore non‐precious metal‐based SACs (such as Fe, Co, Ni, Cu) and reduce costs through strong anchoring strategies to promote large‐scale industrial application. To achieve industrialization, it is also necessary to build continuous flow synthesis platforms that couple atomic layer deposition or electrochemical replacement with continuous flow reactors, enabling large‐scale, reproducible single‐atom production. Additionally, developing online regeneration technologies is crucial to extending the lifespan of the catalyst. Through the "bottom‐up" precise design and regulation of active sites, single‐atom catalysts are expected to become the core of next‐generation efficient and highly selective CO_2_ conversion catalysts, driving breakthroughs in green chemistry and sustainable energy technologies.

## 8 Emerging Trends in Electrocatalysis

**FIGURE 11:**
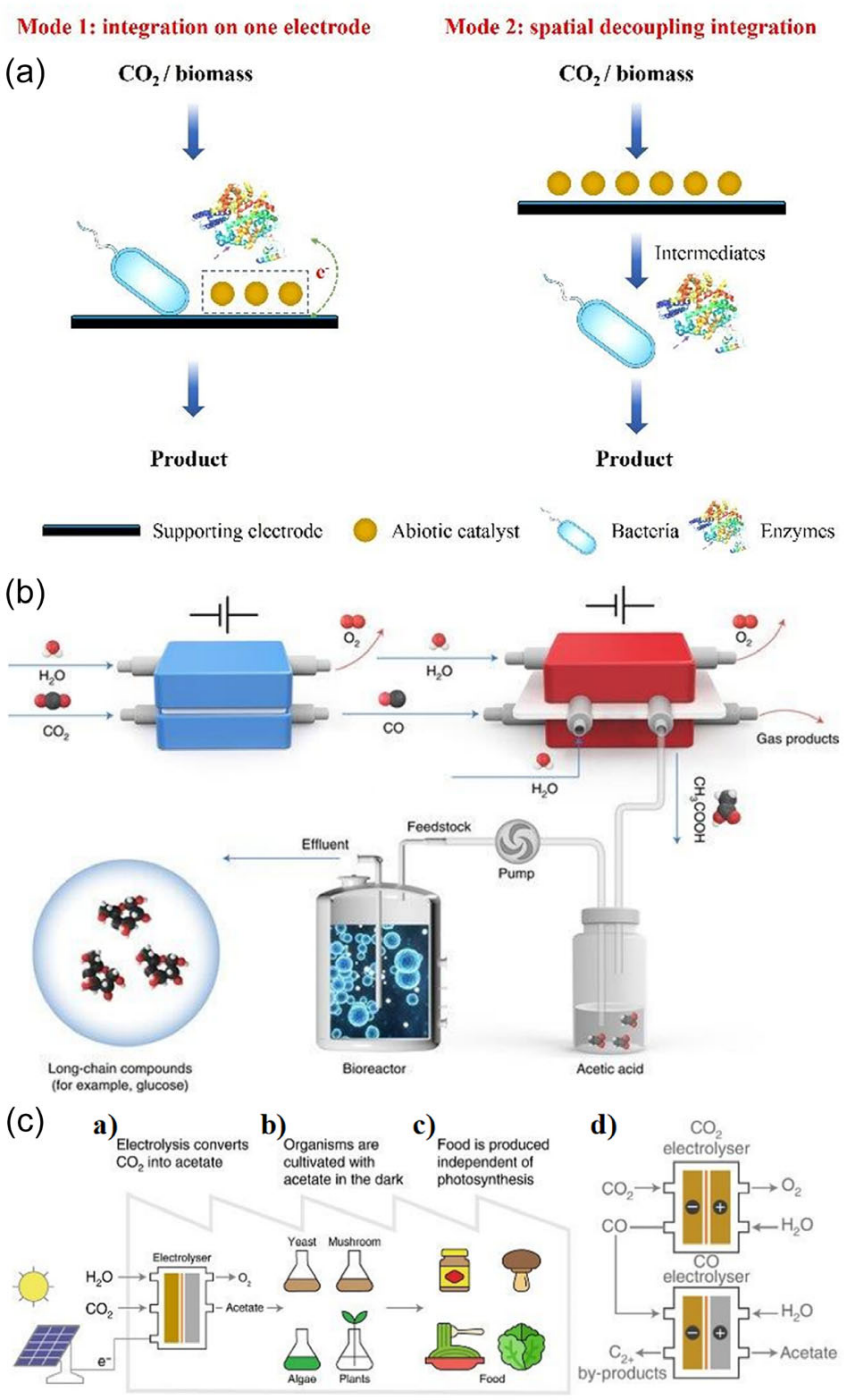
(a) Integrative electrocatalysis and biological synthesis with two modes [125]. (b) The in vitro artificial sugar synthesis combining electrocatalysis and biological catalysis [125]. (c) A combined electrochemical–biological system to produce food from CO_2_ [125]. Reproduced with permission. Adapted from [125]. Copyright 2023, Royal Society of Chemistry.

Electrocatalytic CO_2_ reduction technology uses electricity generated from renewable energy to convert CO_2_ into high‐value chemicals and fuels under mild conditions, providing a green and sustainable innovative pathway for achieving carbon cycling (Figure 11a–c) [125]. As shown in Figure 11a–c, this pathway can achieve upgraded utilization through a "electrocatalysis‐biosynthesis" coupling mode: first, CO_2_ is efficiently electroreduced to C_2_ platform molecules such as acetate, then under dark conditions, these molecules are converted into food components like sugars and proteins via microbial or enzymatic catalysis, thereby bypassing traditional photosynthesis and establishing a closed‐loop system of "CO_2_ → electrocatalytic intermediates → biomanufacturing → food", offering a new paradigm for distributed, land‐independent food production [125]. In recent years, this field has made significant progress in fundamental theory, material innovation, and mechanism research.

### 8.1 Electrochemical CO_2_ Reduction (ECO_2_R)

The core advantage of the electrocatalytic process lies in its reaction rate and product distribution, which can be precisely controlled through the applied potential. It can also be directly coupled with intermittent renewable energy sources, such as solar and wind energy. Current research mainly focuses on catalyst design and engineering.

#### 8.1.1 Nanostructured Electrode

In the study of electrochemical CO_2_ reduction, designing nanostructured electrodes is one of the key strategies to enhance the selectivity and efficiency of C_2_^+^ products [87]. Its core lies in constructing a high specific surface area and abundant active sites through three‐dimensional porosity, core–shell structures, or defect engineering, while significantly enhancing the mass transfer efficiency of CO_2_ [31]. Among them, the copper nanowire arrays are a typical successful example, whose surface is rich in step sites that can significantly enhance the probability of C—C coupling, thereby efficiently promoting the generation of C_2_^+^ products, achieving a Faradaic efficiency of 53.5% for ethylene and a total Faradaic efficiency as high as 87.5% for C_2_^+^ products [152]. In defect‐engineered Cu nanowires, the ability to capture and convert CO intermediates is further enhanced. Moreover, multi‐shell copper nanostructures also show tremendous potential; their unique hollow multi‐shells can effectively increase the local CO concentration through a spatial confinement effect, thereby maintaining an excellent C_2_^+^ product selectivity of over 80% even at industrial‐level current densities as high as 900 mA cm^−2^.

#### 8.1.2 Hybrid Materials (Molecular Catalysts, Conductive Supports)

**FIGURE 12:**
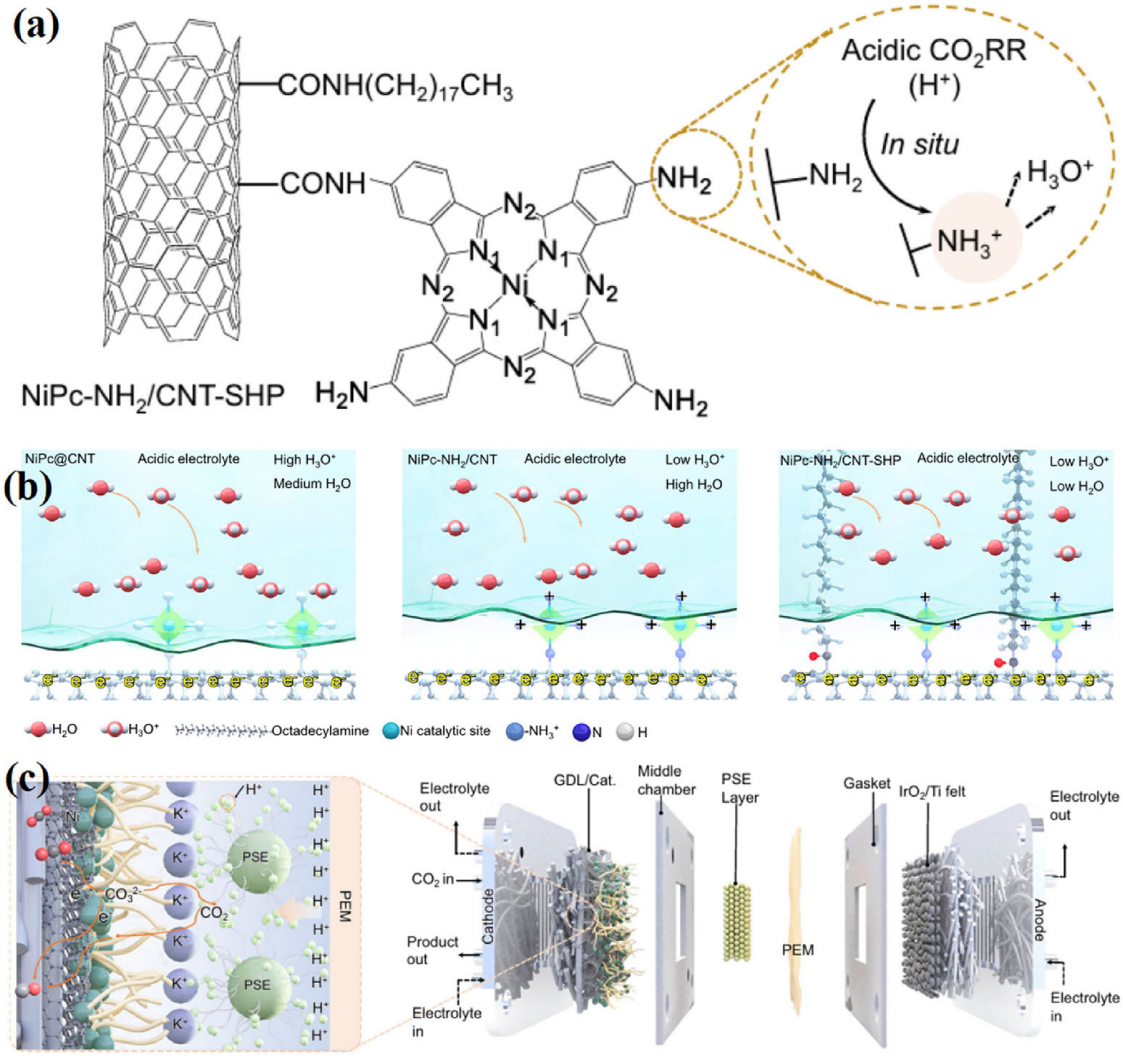
(a) Mechanism diagram of the NiPc‐NH_2_/CNT‐SHP catalyst [236]. (b) Water species distribution on the surface of NiPc@CNT, NiPc‐NH_2_/CNT, and NiPc‐NH_2_/CNT‐SHP [236]. (c) Reducing CO_2_ to CO in our acid PSE reactor [236]. Reproduced with permission. Adapted from [236]. Copyright 2025, Nature Publishing Group.

Hybrid materials significantly enhance the performance and stability of carbon dioxide electroreduction by combining molecular catalysts with conductive supports (Figure 12a–c) [196, 236, 237]. As shown in Figure 12a, in the NiPc‐NH_2_/CNT‐SHP system, the phthalocyanine nickel active centers are covalently anchored onto carbon nanotubes and incorporated with a self‐healing polymer (SHP), which ensures rapid electron transfer while preventing the loss of active sites. Figure 12b further reveals that this surface engineering regulates the distribution of water species, enriches *COOH, and suppresses HER. Figure 12c demonstrates efficient CO_2_‐to‐CO conversion under pH≈1 conditions in an acid proton‐selective electrolyzer (PSE), verifying that the "molecule–conductive support–interfacial water management" synergistic strategy can maintain high activity and long operational stability even under strong acidic conditions [196, 236]. Taking cobalt phthalocyanine (CoPc) anchored on carbon nanotubes (CNT) as an example, this composite structure exhibits excellent CO selectivity in an H‐type electrolytic cell, with a Faradaic efficiency of 92% [191]. In a flow battery with superior mass transfer, its CO selectivity is further increased to 99%. Furthermore, by constructing a synergistic system of CoPc and Cu nanoparticles (Cu/CoPc), the CoPc sites can efficiently generate a large amount of CO intermediates, which then "overflow" to the neighboring Cu sites, significantly promoting the C—C coupling process and increasing the Faradaic efficiency of C_2_^+^ products to 73%. In addition, the material constructed by combining CoPc with graphene also demonstrates excellent adaptability in a simulated biogas environment with low CO_2_ concentration (around 40%), achieving a CO Faradaic efficiency of 93.7%. At the same time, the reaction voltage decreases by about 1 V, showing potential for practical applications under complex gas source conditions.

#### 8.1.3 Membrane Electrode Assembly (MEA) Innovation

**FIGURE 13:**
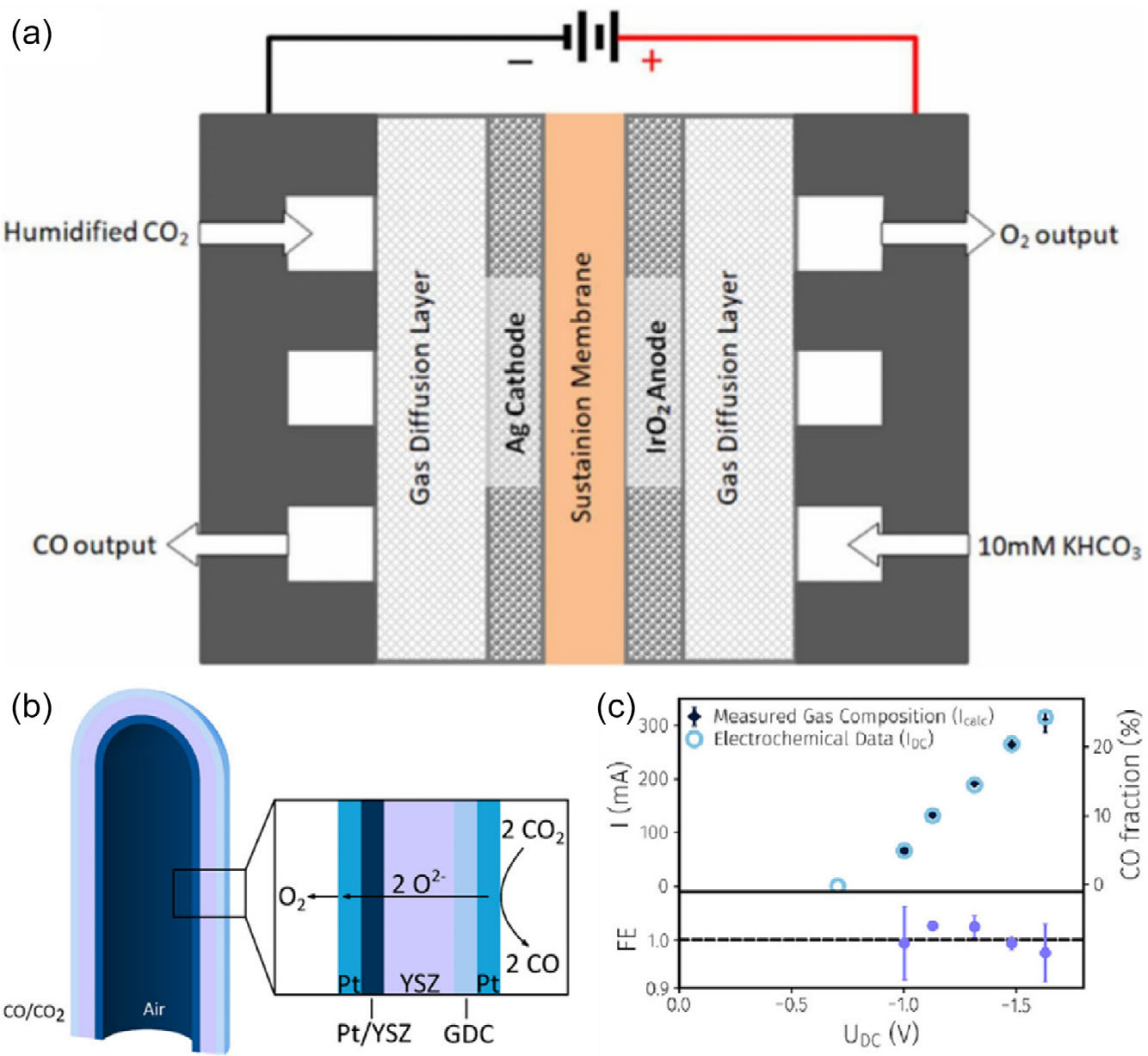
(a) Principle of CO_2_ electrolyzer [153]. Reproduced with permission. Adapted from [153]. Copyright 2025, MDPI. (b) Sketched cross‐section of a tubular SOEC consisting of a GDC cathode, Pt/YSZ anode, and YSZ electrolyte, which was used for online coupling CO production via high‐temperature CO_2_ electrolysis with carbonylation reactions [238]. (c) Current–voltage curve (IDC vs. UDC) together with the current Icalc obtained from the measurement of the CO concentration in the product gas for a flow of 10 mL CO_2_ min^−1^ [238]. Reproduced with permission. Adapted from [238]. Copyright 2025, Wiley‐VCH GmbH.

In the field of electrochemical CO_2_ reduction, innovations in membrane electrode systems are the core driving force for advancing toward industrial applications (Figure 13a) [153, 239]. Solid electrolyte membrane assemblies, particularly high‐temperature solid oxide electrolyzers, can directly electrolyze CO_2_ into dry CO_2_ and couple this process with downstream carbonylation processes to achieve continuous synthesis of high‐value chemicals (Figure 13b,c) [238]. As shown in Figure 13b, the tubular SOEC composed of a GDC cathode, Pt/YSZ anode, and YSZ electrolyte can electrolyze CO_2_ into pure CO at high temperatures and immediately feed it into an online carbonylation reactor. Meanwhile, the current–voltage curve in Figure 13c and the I_calc_ calculated based on the CO concentration of the product gas, according to Faraday's law, indicate that under a CO_2_ feed of 10 mL min^−1^, the electrolysis current corresponds closely with the CO yield. The faradaic efficiency (FE) rapidly approaches 100% as the applied voltage increases, confirming the advantages in both efficiency and selectivity of the integrated process of “high‐temperature SOEC → dry CO → in situ carbonylation” [238]. In terms of system scaling, zero‐gap MEAs demonstrate tremendous potential. For example, a catalyst based on three‐dimensional single‐atom Ni‐N_3_ achieved a CO Faradaic efficiency of 98.9% in a 2 × 2 cm^2^ MEA and maintained over 97% CO selectivity in a 10 × 10 cm^2^ scaled‐up device, with a single‐channel CO_2_ conversion rate as high as 41%. Structural innovation is equally crucial. A support‐free silver‐based MEA, by directly transferring the catalyst layer onto the ion exchange membrane and eliminating the traditional gas diffusion layer, not only significantly reduces cell voltage but also enhances CO selectivity [240]. The synergistic optimization of operating strategies and membrane materials has also been remarkable: combining pulse electrolysis with Sustainion ion membranes in zero‐gap MEAs can nearly double the Faradaic efficiency of multi‐carbon products at current densities above 300 mA cm^−2^, which is mainly attributed to the effective enhancement of local CO_2_ concentration by the pulsing strategy [141, 241]. In addition, by modifying cation exchange membranes, such as using the positively charged ion conductor PiperION to modify their surface to create a favorable cathode microenvironment, the Faradaic efficiency of formic acid can reach 80% at 300 mA cm^−2^. At the same time, the operating voltage is reduced by about 1 V compared to traditional bilayer membrane structures [231].

CO_2_ electroreduction technology is rapidly developing towards systematization and practical application. From a comprehensive review, current research has shown a diversification of control methods: from the design of defects/porous structures at the material level, synergistic catalysis of molecules and metals, to the regulation of potential, pulse waveforms, and electrolyte ion types at the cell level, all can achieve precise guidance of reaction pathways, thereby optimizing product distribution [242]. At the same time, this technology is steadily advancing toward continuous and large‐scale operation. Systems based on solid‐state membrane electrolysis, zero‐gap MEAs, and single‐atom/multi‐atom catalysts have achieved continuous and stable operation at tens to hundreds of amperes on a laboratory scale, preliminarily validating feasible commercialization pathways. In addition, its natural integration with renewable energy also shows broad prospects: the electrocatalytic process's direct dependence on electricity allows it to quickly respond to fluctuating power sources, such as solar and wind energy, effectively balancing the grid load and thereby improving the overall efficiency of future renewable energy systems [243].

Looking to the future, research prospects in this field focus on several key directions. First, it is essential to further enhance the selectivity and efficiency of C_2_^+^ products, which relies on combining in situ spectroscopy with computational chemistry for joint mechanistic analysis and catalyst design, to achieve precise control over the adsorption and transformation behaviors of key intermediates such as CO and OCCO [244, 245]. Second, reducing system energy consumption is key to economic feasibility, with a focus on developing solid electrolytes and ion membranes that possess high ionic conductivity and low Ohmic resistance, enabling stable operation at low voltage under high current, while continuously optimizing their long‐term stability and cost [93, 246]. The ultimate goal is to achieve full‐chain carbon cycle integration, combining CO_2_ capture, electrochemical conversion, product separation, and subsequent high‐value chemical synthesis (such as biotransformation) into an integrated closed‐loop system, thereby truly establishing a sustainable carbon‐neutral technology pathway [196].

### 8.2 Reaction Mechanism

The following discussion on key intermediates and reaction mechanisms pertains specifically to the electrocatalytic CO_2_ reduction pathway [113]. The intermediates (COOH, CO, CHO, etc.) and their evolution are governed by the applied potential and occur at the electrochemical interface [247]. This mechanistic picture is distinct from that in thermocatalytic hydrogenation, where the formation and transformation of intermediates (e.g., formate, methoxy) are driven by thermal energy and surface hydrogenation steps, leading to different product spectra (e.g., high selectivity for methanol via the formate pathway in thermocatalysis versus a mix of CO, hydrocarbons, and oxygenates on Cu in electrocatalysis).

**FIGURE 14:**
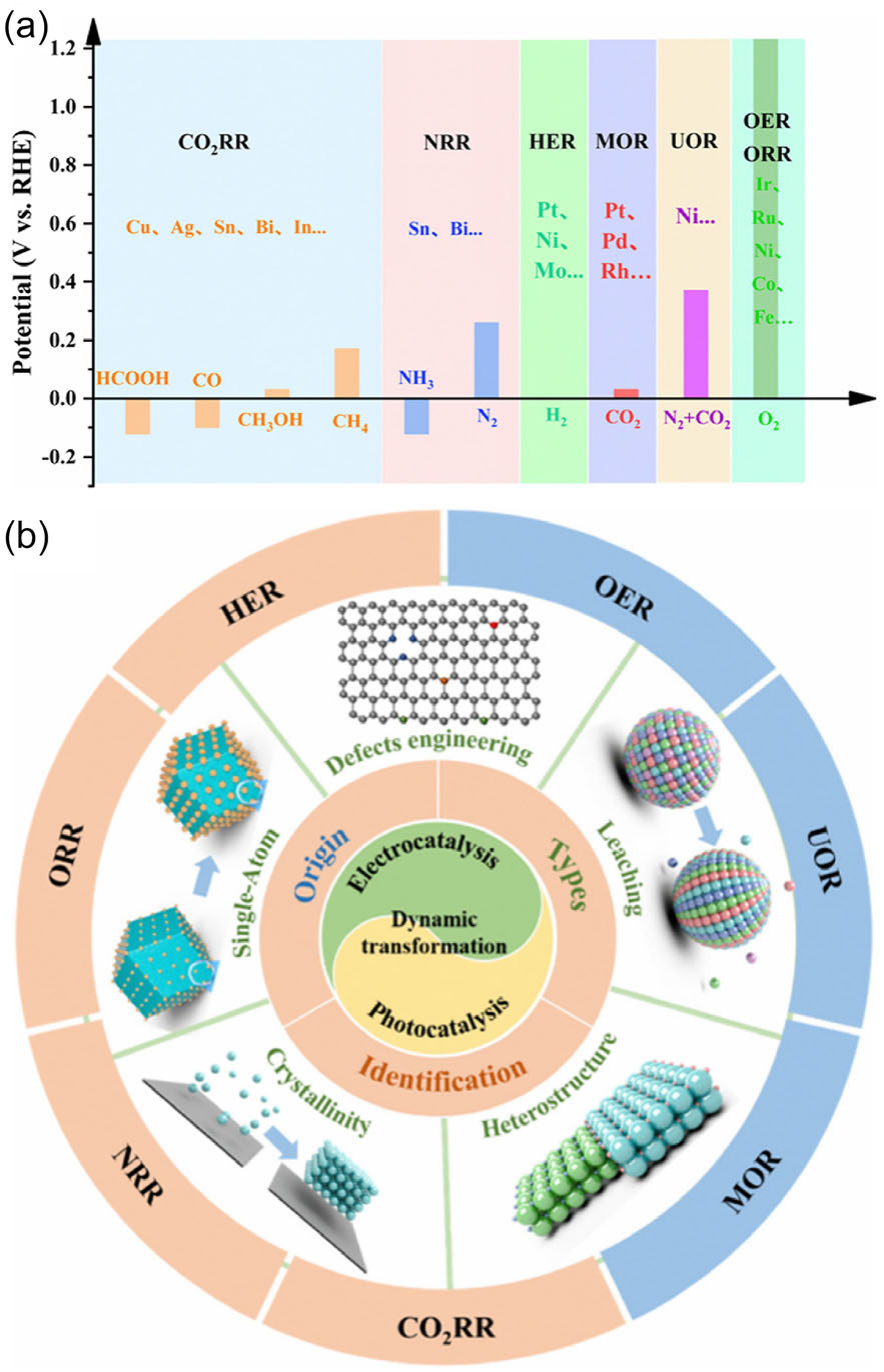
(a) Electrocatalytic reactions and the related standard equilibrium potentials versus the reversible hydrogen electrode (RHE) under standard conditions [248]. (b) Dynamic transformation of active sites in the field of photo/electrocatalysis, covering the origin and types of dynamic transformation, identification methods, various triggering factors, and applications in photo/electrocatalysis [248]. Reproduced with permission. Adapted from [248], Copyright 2024, Royal Society of Chemistry .

CO_2_ reduction (ECO_2_RR) is a series of processes occurring continuously on the electrode surface, including adsorption, activation, hydrogenation, and desorption (Figure 14a) [248, 249]. The binding energy, coverage, as well as changes in the local electric field, pH, and ionic environment of intermediates at each step, directly determine the selectivity and overall activity of the products [250]. Therefore, placing the real‐time revelation of dynamic reaction pathways at the core of catalyst design is key to achieving efficient and selective conversion (Figure 14b) [70, 147, 248].

#### 8.2.1 Identification and Role of Key Intermediates

**FIGURE 15:**
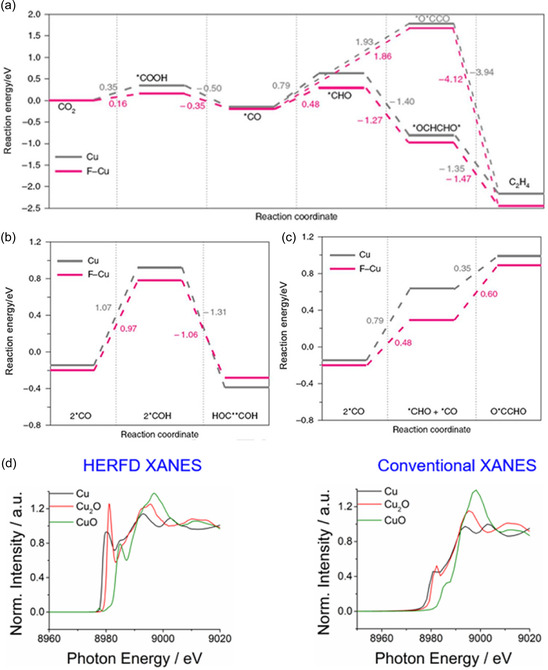
(a) Reaction energy diagram for the CO_2_RR to C_2_H_4_ on Cu(111) and F‐Cu(111) facets, either via a direct *CO dimerization pathway or *CO hydrogenation to *CHO followed by a dimerization pathway [246]. (b) Reaction energy diagram for *CO hydrogenation to *COH and subsequent dimerization to HOC**COH on Cu(111) and F‐Cu(111) facets [246]. (c) Reaction energy diagram for *CO hydrogenation to *CHO and subsequent coupling with another *CO to O^*^CCHO on Cu(111) and F‐Cu(111) facets [246]. Reproduced with permission. Adapted from [246]. Copyright 2022, Springer Nature. (d) Comparison of different copper standards measured in HERFD and conventional XANES modes [213]. Reproduced with permission. Adapted from [213]. Copyright 2023, American Chemical Society.

On copper‐based electrodes, *COOH and *CO are the two key intermediates that determine product branching (Figure 15a–c) [246]. As shown in Figure 15a, on the Cu(111) and F‐Cu(111) surfaces, *CO can either dimerize directly or first be hydrogenated to *CHO and then couple. The difference in energy barriers of these two pathways determines the ethylene preference. Figure 15b,c further reveals the detailed energy barrier diagrams for the hydrogenation of *CO to *COH, followed by the formation of HOC**COH, or hydrogenation to *CHO, followed by coupling with another CO to form OCCHO. This confirms that fluorine modification significantly lowers the energy barriers for *CO protonation and C—C coupling, thereby achieving precise switching of product selectivity by regulating the relative stability of *CO/*COH/*CHO [246]. Density functional theory (DFT) calculations, combined with isotope labeling experiments, indicate that the coverage of *CO and the degree of hydrogenation directly control the formation pathways of alkanes and alcohols [251]. The adsorption energy of *COOH varies significantly on different crystal faces of copper. Cu(110), due to its highest d‐band center, can most effectively facilitate the conversion of *COOH → *CO, making it a potential active site for the formation of multi‐electron products, such as methanol [252]. In addition, C_2_ intermediates such as *OCCO and *CH_2_CHO are enhanced at the Cu(100)/Cu(111) interface, where these interfaces provide *CO sites with varying binding strengths, significantly improving the efficiency of C—C bond coupling [183].

#### 8.2.2 Breakthroughs in In Situ/Operando Characterization

##### 8.2.2.1 X‐Ray Absorption Spectroscopy (XAS)

High‐energy resolution fluorescence detected XAS (HERFD‐XAS) can track changes in the oxidation state and coordination number of Cu nanoparticles in real time under working potential, revealing that metallic Cu^0^ nanocrystals are the actual active sites. At the same time, the surface Cu^+^/Cu^0^ coexisting oxidized‐metal matrix (MEOM) model further explains the synergistic effect of CO_2_ activation and CO dimerization (Figure 15d) [167, 213].

##### 8.2.2.2 Surface‐Enhanced Raman Spectroscopy (SERS/SHINERS)

In Raman cavities enhanced by Au@SiO_2_ nanoparticles, it is possible to capture the characteristic peaks of intermediates such as *CO_2_^‐^, *COOH, and *CO at the atomic scale, clarifying the distribution of intermediates in different potential regions.

##### 8.2.2.3 Online Mass Spectrometry (DEMS)

Through synchronous detection of gas and liquid phases, instantaneous quantification of products such as CO, C_2_H_4_, and ethanol is achieved, providing experimental evidence for constructing dynamic activity maps [253].

##### 8.2.2.4 In‐Situ X‐Ray Microscopy (EC‐TEM)

Real‐time observation of the morphological evolution of Cu nanostructures in an electrochemical flow cell, confirming that at high current densities, Cu nanoparticles undergo roughening and local reconstruction, leading to *CO poisoning and affecting long‐term stability [254, 255].

#### 8.2.3 The Latest Understanding of Microenvironment Effects

##### 8.2.3.1 Local pH

At high current densities (> 150 mA cm^−2^), the accumulation of OH^‐^ on the electrode surface can raise the local pH by up to 5 units compared to the bulk phase, significantly suppressing the hydrogen evolution reaction (HER) while enhancing the selectivity for C_2_^+^ products [59, 256].

##### 8.2.3.2 Electric Field and Cation Distribution

The accumulation of K^+^ at the electrode/electrolyte interface can reduce the desorption energy of *CO and promote CO—CO coupling [257]. However, its solubility limit (≈3.5 M) restricts the K^+^ concentration in conventional electrolytes. By using a Cu nanoneedle structure to achieve local K^+^ supersaturation (4.22 M), the solubility limit is overcome, significantly enhancing the Faradaic efficiency for C_2_^+^ to over 90% [211].

##### 8.2.3.3 Ionic Aggregation State

In alkaline electrolytes, the interaction between K^+^ and the Cu surface creates an electric field gradient, which regulates *CO coverage and thus affects the rate of C—C bond formation [258].

##### 8.2.3.4 Dual‐Site Synergy

The in‐situ construction of dual atomic sites with metals such as Co, Zn, and Sn on Cu (e.g., Cu—Co, Cu—Sn, Cu—Zn) can provide CO_2_^‐^ activation sites and CO—CO coupling sites on the same surface, achieving high selectivity for C_2_^+^ production at low overpotentials [199]. For example, Cu—Co dual sites in a g‐C_3_N_4_ framework can enhance CO adsorption through electronic asymmetry and reduce the C—C coupling barrier, with FE(C_2_^+^) exceeding 60 % [259].

##### 8.2.3.5 Single‐Atom/Defect Engineering

Single‐atom or bimetallic sites, such as Ni—P, Ni—Fe, and Ni—Sn, significantly reduce the formation free energy of *COOH by modulating the d‐band center, thereby achieving nearly 100% CO selectivity over a wide potential window [260]. Sn single atoms embedded in the CuO matrix (Sn^4+^—O_3_—Cu^+^) show regulation of the *COOH adsorption configuration under operando Mössbauer and XAS monitoring, enhancing the CO_2_→CO conversion efficiency to 98% [261].

#### 8.2.4 The Concept of Rational Catalyst Design

##### 8.2.4.1 Dual‐Site/Multi‐Metal Synergy

Construct in situ dual‐atomic sites of Cu with metals such as Co, Zn, and Sn on the same support, allowing both *CO_2_^‐^ and *CO—CO activation centers to coexist [262]. Experiments show that this type of structure can achieve high selectivity for C_2_^+^ products at low overpotentials, while the stability of intermetallic electron transfer is verified through operando XAS and SERS [263].

##### 8.2.4.2 Single‐Atom Ligand Regulation

Utilizing ligands such as P, N, and O to achieve axial coordination of single atoms like Ni, Co, and Sn (e.g., Ni—P, Ni—N_4_—P), significantly enhancing the adsorption strength of *COOH and suppressing HER, achieving a CO Faradaic efficiency above 99% [264].

##### 8.2.4.3 Defects and Oxygen Vacancies

**FIGURE 16:**
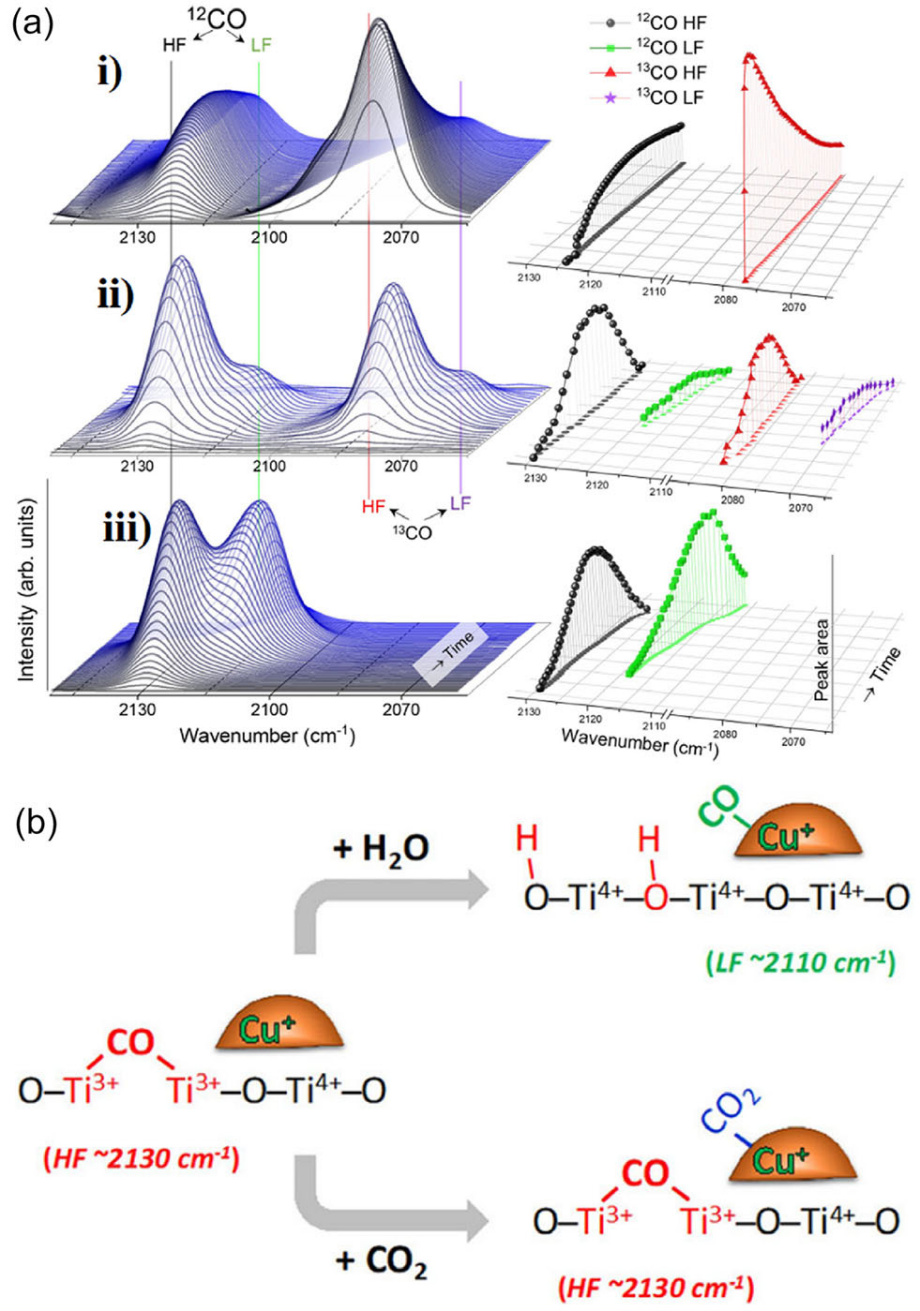
(a) ^13^CO_2_ isotope labeling experiment. DRIFTS spectra of surface‐adsorbed CO as a function of time on Cu/TiO_2_ samples pretreated at 300°C under Ar flow [265]. (b) Competitive adsorption of CO with H_2_O and CO_2_ [265]. Reproduced with permission. Adapted from [265]. Copyright 2022, Springer Nature.

Introducing oxygen vacancies in Cu/TiO_2_ and CuO‐based systems enables CO_2_ to bind at dual sites between Cu and oxygen ions, resulting in a lower activation energy and promoting the formation of *CO and subsequent C—C coupling (Figure 16a,b) [265, 266, 267].

##### 8.2.4.4 Microenvironment Engineering

Achieving local K^+^ supersaturation through carbon shells or nanoneedle structures, regulating the electric field and local pH, significantly enhancing C_2_^+^ yield [211, 268]. Simultaneously design hydrophilic/hydrophobic hierarchical structures in gas diffusion electrodes (GDEs) to prevent electrolyte flooding and maintain the stability of the three‐phase interface, thereby achieving over 64% C_2_^+^ selectivity even at industrial current densities above 300  mA  cm^−2^.

##### 8.2.4.5 Multi‐Scale Closed‐Loop Verification

From DFT calculations predicting free energy curves → synthesis of target structures → real‐time monitoring with operando XAS, SERS, and DEMS → cross‐validation of structure–activity relationships [269]. The design is considered successful only when all three types of representations demonstrate that the target site remains stable under working potential, and the product distribution is consistent with calculations [270].

#### 8.2.5 Prospects and Challenges

CO_2_ reduction technology still faces a series of challenges and opportunities as it moves from the laboratory to industrialization. First, the combination of high‐throughput screening and machine learning will significantly accelerate material development [271, 272]. By leveraging dynamic databases constructed from operando characterization techniques (such as XAS, SERS, DEMS), models capable of accurately predicting the catalytic activity of dual‐site or defect structures can be trained, thereby significantly shortening the experimental trial‐and‐error cycle.

However, on the road to industrialization, microenvironment control and long‐term stability are two major bottlenecks. Under industrial‐level high current density conditions (> 1 A cm^−2^), maintaining uniformity of local pH and ion concentration on the electrode surface is a significant challenge, which urgently requires innovations in fluid dynamics and electrode structure design. At the same time, the issue of long‐term operational stability is increasingly prominent. Operando X‐ray scattering and other techniques have revealed that metal copper catalysts can suffer from CO intermediate poisoning due to surface roughening under high current conditions [167]. To address this issue, constructing a stable oxide–metal matrix on the copper surface or alloying (such as Cu—Ga) can effectively suppress its irreversible redox cycles, thereby significantly extending the catalyst's lifespan [273].

Another promising direction is photoelectrochemical co‐catalysis. By cleverly combining a photocatalyst with electrochemical dual sites, the synergistic advantages of photogenerated electrons and an applied electric field can be leveraged to achieve higher energy utilization efficiency and product selectivity [84]. For example, Cuδ/CeO_2_—TiO_2_‐based systems have achieved 73% selectivity for C_2_^+^ products in the laboratory, laying a solid foundation for the future development of large‐scale photoelectrochemical reduction devices [274].

Therefore, by capturing reaction intermediates in situ, precisely regulating the reaction microenvironment, and synergistically designing dual sites and defect structures, the performance limits of CO_2_ reduction have been approached at the laboratory level [252, 275, 276]. The next key step is to transform these "laboratory‐level" breakthrough strategies into scalable and economically sustainable industrial processes. Achieving this goal ultimately depends on the deep, closed‐loop collaboration of multiple disciplines, including materials science, in situ characterization, fluid dynamics, and process engineering.

### 8.3 Key Materials

In the field of electrocatalytic CO_2_ reduction, MXenes, doped graphene, and metal–organic frameworks are the core pillars of current electrode development due to their unique structures and properties [124, 277].

**FIGURE 17:**
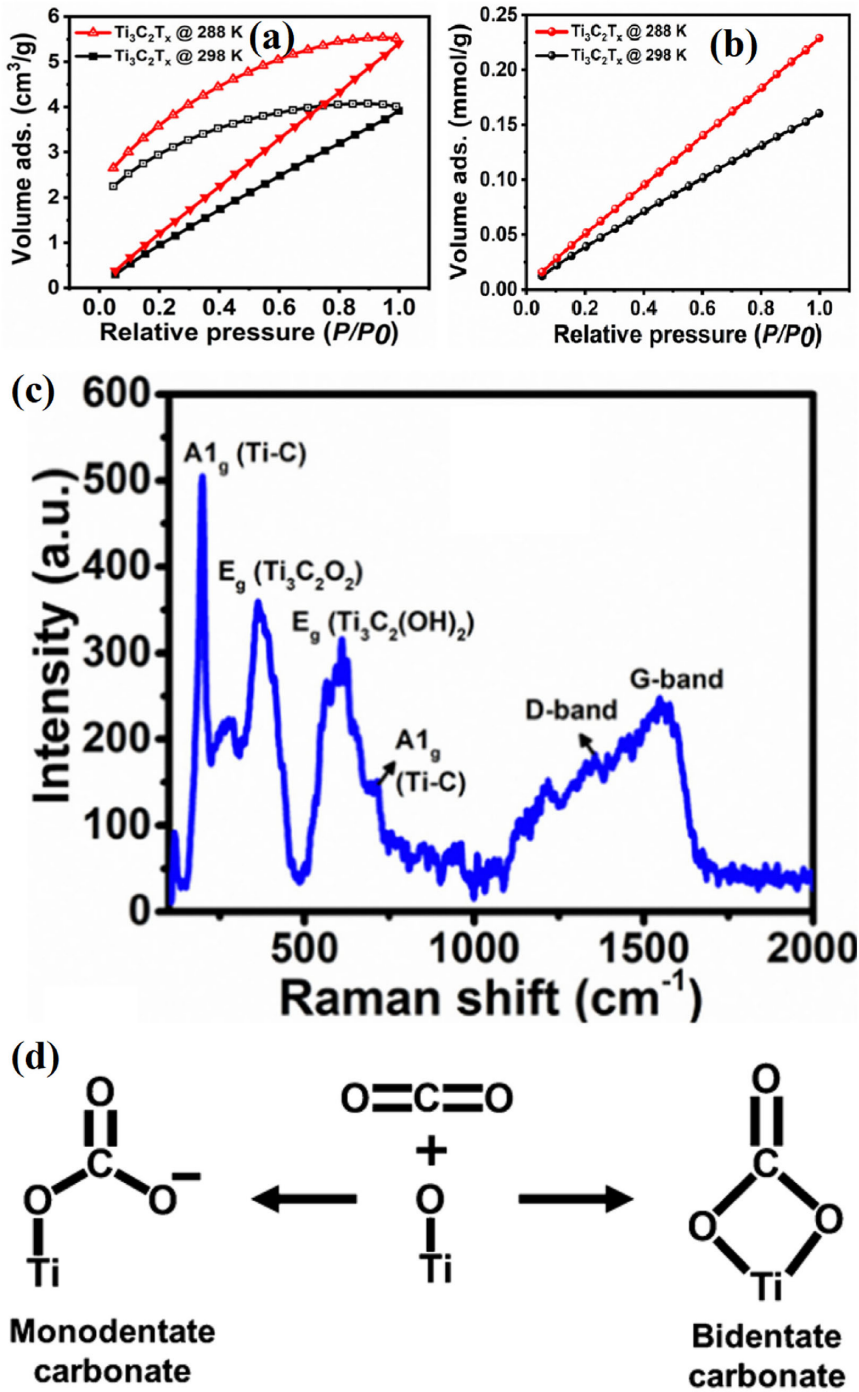
(a,b) CO_2_ adsorption–desorption isotherm in cm^3^ g^−1^ and CO_2_ adsorption isotherm in mmol g^−1^ at 288 and 298 K [278]. (c) Raman spectrum [278]. (d) Possible CO_2_ interaction on the MXene surface [278]. Reproduced with permission. Adapted from [278]. Copyright 2023, Royal Society of Chemistry.

MXenes, especially Ti_3_C_2_T_*x*_, exhibit outstanding CO_2_ activation capabilities due to their inherent high conductivity and surface‐tunable functional groups (such as —O, —OH) [278]. As shown in Figure 17a,b, this material exhibits reversible CO_2_ adsorption–desorption isotherms at 288–298 K, with adsorption amounts on the order of mmol g^−1^, confirming that its oxygen‐rich surface can provide ample Lewis acid sites. The Raman spectra in Figure 17c further show shifts in the characteristic peaks of adsorbed CO_2_, indicating molecular bending activation, while the "CO_2_ forming bidentate carbonate with surface —O groups" model proposed in Figure 17d intuitively explains the mechanism by which MXene weakens the C=O bond through reversible charge transfer, reducing subsequent protonation barriers, providing the dual advantages of a highly conductive substrate and a tunable chemical microenvironment for efficient electrochemical CO_2_ reduction [278]. These hydrophilic groups can form strong interactions with CO_2_ molecules, significantly lowering the activation energy barrier [89]. For example, Ti_3_C_2_T_*x*_ anchored by S, in synergy with Zn, can achieve a Faradaic efficiency of approximately 89.7% for CO [279]. The two‐dimensional layered structure offers a large specific surface area and abundant edge sites, facilitating the loading of metal nanoparticles, such as Pt and Ni [280]. When these particles combine with N‐doped MXene substrates, they can further accelerate electron transport, maintaining low overpotential and long‐term stability even in harsh electrolytes [281].

Doping with graphene, especially types doped with heteroatoms such as N and S, enhances performance by modulating the electronic structure of carbon materials [94, 282, 283]. Pyridinic nitrogen sites in nitrogen‐doped graphene can disrupt the electronic symmetry of the carbon framework, creating electron‐rich centers that selectively convert CO_2_ into CO [21, 284]. N, S co‐doped materials can exhibit a synergistic effect more effectively: S enhances the polarization of the carbon framework to stabilize the COOH intermediate, while N promotes the desorption of CO products. Together, they synergistically achieve a Faradaic efficiency of about 80% at low overpotential [51]. This type of material is easy to prepare and can be produced on a large scale at low cost through thermal treatment of graphene oxide or by using sulfur‐containing precursors. Its metal‐free and abundant nature, coupled with the ability to control product distribution through precise doping, gives it enormous application potential.

Metal–organic frameworks (MOFs) and their derivatives provide a highly designable platform. When used directly as catalysts, the ordered pores of MOFs can achieve confined enrichment of CO_2_; however, their poor intrinsic conductivity is a major bottleneck, necessitating improvement by preparing ultrathin nanosheets or compositing with carbon nanotubes [285, 286]. More research focuses on MOF derivatives, especially single‐atom/metal‐carbon composites obtained by pyrolysis. For example, Fe—N—C catalysts prepared from pyrolyzed ZIF‐8 precursors exhibit a Faradaic efficiency for CO of nearly 100% [287]. Embedding Ag nanoparticles into the M‐N/Al‐O framework derived from MOF‐253(Al) can stabilize key intermediates through electron density reconstruction, achieving a CO efficiency of 85.6% [31]. By introducing a secondary carbon source into the MOF precursor, the pore structure of the derived material can be finely tuned, significantly enhancing the accessibility of CO_2_ and achieving a CO Faradaic efficiency of up to 95% at −0.50 V versus RHE [288, 289].

In terms of overall outlook and development pathways, although breakthroughs have been made in active site density (such as single‐atom sites), electron transport (such as MXene‐based matrices), and product selectivity (such as N, S‐doped graphene), challenges such as large‐scale homogeneous preparation, interface impedance control at high current, and high selectivity for C_2_^+^ products remain critical bottlenecks. Future technical routes will rely on the combination of AI‐driven high‐throughput screening and in situ characterization for precise catalyst design. At the same time, multi‐scale interface engineering (such as constructing MXene‐metal‐carbon ternary composites), promoting C—C coupling through synergistic metal sites or local microenvironment regulation, and developing continuous‐flow electrolyzers and low‐cost precursors are key to improving industrial feasibility.

Therefore, MXenes, doped graphene, and MOF‐derived materials have collectively advanced ECO_2_R technology by enhancing conductivity, regulating surface chemistry, and creating high‐density active sites [290]. The key to future breakthroughs lies in achieving multi‐scale synergistic design of “catalyst‐interface‐reactor” and deeply integrating AI‐assisted experiments with in situ characterization techniques, thereby accelerating the transformation of high‐performance materials from laboratory discovery to industrial application.

## 9 Industrial Applications and Upscaling

The transition of CO_2_ catalytic conversion from fundamental research to industrial application presents distinct pathways and challenges that are intrinsically linked to the underlying technology [291]. This chapter discusses scaling efforts by explicitly distinguishing between the thermocatalytic and electrocatalyticroutes towards industrialization. The pilot projects, core challenges, and system integration strategies differ fundamentally between a centralized, high‐temperature thermochemical plant (e.g., for methanol synthesis) and a modular, electricity‐driven electrochemical process (e.g., for CO or ethylene production). The following analysis will maintain this pathway‐specific perspective to provide a clear and logical assessment of the industrialization landscape [164].

Scaling up CO_2_ catalytic conversion technology from the laboratory to industrialization is a crucial step in realizing its climate and economic value [292]. Currently, this field is undergoing a significant transition from basic research to pilot‐scale demonstration, with multiple technological routes showing promise for industrial application.

### 9.1 Pilot Projects

Worldwide, several pilot projects are dedicated to transitioning CO_2_ conversion technologies from the laboratory to commercialization [293, 294]. Among them, Carbon Recycling International (CRI) operates the “George Olah” plant in Iceland as a pioneer. It employs a thermocatalytic route, utilizing Cu/ZnO/Al_2_O_3_ as the catalyst, to synthesize methanol from CO_2_ and renewable hydrogen generated from geothermal electricity. The plant has an annual output of approximately 4000 tons and has successfully entered the chemical fuel market, verifying the continuous operational reliability and preliminary economic feasibility of the process [295, 296]. In Germany and Northern Europe, the collaboration between Sunfire and Nordic Chemicals explores another approach: producing hydrogen through high‐temperature electrolysis, then converting CO_2_ into CO via the reverse water–gas shift (RWGS) reaction, and further synthesizing sustainable aviation fuel through the Fischer–Tropsch process. The key innovation lies in the efficient integration of high‐temperature electrolysis with the RWGS reaction [297, 298]. At the same time, Canada's Carbon Engineering has developed a pilot electrochemical reduction platform capable of converting CO_2_ into syngas with an adjustable H_2_/CO ratio, directly providing feedstock for downstream Fischer–Tropsch synthesis or methanol production, and combining it with direct air capture technology [299]. KU Leuven in Belgium focuses on electrochemical membrane reactors, suppressing the hydrogen evolution side reaction through specialized ion‐exchange membranes, achieving highly selective CO production and continuous, stable operation for over 1000 h, demonstrating excellent engineering potential [196, 300].

These pilot projects reveal common key technologies and challenges associated with scale‐up. In terms of hydrogen sources, the projects address the cost and variability issues of renewable hydrogen through geothermal power, high‐temperature electrolysis, or coupling with DAC [296, 301]. In the CO_2_ capture and transportation process, on‐site capture or direct air capture has become a feasible approach to reduce costs [233]. In terms of catalyst stability, the mature Cu/ZnO/Al_2_O_3_ system demonstrates robustness at moderate temperatures, while new electrocatalysts such as PdH and Ni—N—C are also under continuous development [302]. To achieve a flexible adjustment of the syngas ratio, the electrochemical approach offers unique advantages by adjusting the current density or selecting different electrode materials [303]. In the core challenge of system integration, zero‐gap membrane electrodes, high‐temperature heat recovery, and modular design are effective strategies for improving overall energy efficiency.

These advances are closely tied to collaboration among industry, academia, and research institutions. University laboratories provide theoretical support in cutting‐edge areas, such as catalyst mechanisms, membrane materials, and reaction kinetics. Enterprises, on the other hand, lead process engineering scale‐up and equipment manufacturing to ensure the reliability of technology, from laboratory experiments to pilot tests and even megawatt‐scale operations. Additionally, low‐carbon policies and financial support from governments worldwide provide critical momentum for technological innovation and market access.

Despite the broad prospects, some information gaps still need to be filled, such as the detailed operational parameters of Carbon Engineering's facility and the complete economic assessment data for the Sunfire and Nordic Chemicals projects, which can be further obtained from the company's technical reports or patents.

Therefore, the current pilot projects provide clear guidance for future commercialization: a modular design facilitates rapid deployment and technological replication [304]. The electrochemical approach has significant advantages in flexibly adjusting the synthesis gas ratio [305]. Thermoelectric coupled systems are an important direction for maximizing energy efficiency [306]. Long‐term, stable operational data (such as over 1000 h at KU Leuven) provides a crucial industrial benchmark for assessing technological maturity and durability [307]. These practices collectively indicate that, through continuous technological optimization and system integration, the commercialization path for CO_2_ resource utilization is becoming increasingly clear [308].

### 9.2 Upscaling Challenges

To scale up CO_2_ conversion technology for practical application, three major core challenges must be overcome: catalyst durability, process efficiency, and system integration, ultimately leading to economic feasibility [184]. This requires us to adopt an interdisciplinary systems engineering approach, rather than addressing a single problem in isolation.

#### 9.2.1 Catalyst Durability: From Deactivation Mechanisms to Long‐Lasting Design

In an industrial amplification environment, catalysts face challenges far more severe than those in the laboratory. The primary deactivation mechanisms include poisoning by impurities such as sulfur, nitrogen, and chlorine in the feed gas, structural damage resulting from thermal shocks, and pulverization due to mechanical wear [309]. Recent research has provided various solutions for this: developing multifunctional alloys or metal–oxide composites to enhance resistance to deactivation [310, 311]. Using layered MOFs or COFs as carriers, stabilizing active sites through their tunable pore sizes and enhanced structural rigidity [312]. Utilizing in situ regeneration strategies, such as temperature pulses or oxidation/reduction cycles, to dynamically restore catalyst activity [191]. To achieve long‐term operation, a systematic solution has emerged:

##### 9.2.1.1 Structured Catalysts

Depositing the active phase onto high‐strength ceramic or metal substrates to form structures with gradient pore sizes or honeycomb patterns, while balancing mass transfer requirements and mechanical strength [194, 313].

##### 9.2.1.2 Online Regeneration System

Integrates a regeneration section within the reactor, utilizing process waste heat or renewable electricity to achieve rapid detoxification and repair [314].

##### 9.2.1.3 Impurity‐Tolerant Formulation

Pre‐add poisons (such as calcium‐based oxides) in the catalyst formulation or perform surface modification to reduce the impact of impurity adsorption from the source [315].

#### 9.2.2 Process Efficiency and System Integration: Overcoming Bottlenecks in Mass Transfer, Heat Transfer, and Energy Coupling

The low efficiency of the CO_2_ conversion process often stems from a series of coupled bottlenecks: the low CO_2_ concentration (10%–15%) leads to high energy consumption for prior capture and concentration; mass transfer limitations during the reaction, and the difficulty of thermal management in exothermic reactions are intertwined [316]. Current research hotspots focus on process intensification, such as utilizing microchannel reactors to leverage their high specific surface area and enhance mass transfer rates, thereby reducing temperature gradients [317]. Design structured beds (e.g., grids, fibers) to enhance fluid distribution and heat recovery. Introduce process intensification techniques, such as fluid oscillation and centrifugal mixing, to overcome transfer limitations. At the system level, the key lies in deep integration:

##### 9.2.2.1 Coupled Capture‐Conversion‐Separation

Integrating amine absorption/membrane separation, high‐pressure conversion, and product separation into a single device or closely coupled process to create a lower‐energy, closed‐loop system [318, 319].

##### 9.2.2.2 Smart Scheduling of Renewable Energy

By utilizing energy storage and demand‐side management, high‐energy‐consuming processes such as electrolysis and electrocatalysis can be coordinated with fluctuating wind and solar power generation to stabilize electricity costs [320].

##### 9.2.2.3 Modular Parallel Amplification

By connecting a large number of microchannel units in parallel, flexible expansion of production capacity is achieved while maintaining ease of maintenance and high transmission efficiency [321].

#### 9.2.3 Economic Feasibility: Building a TEA‐ and LCA‐Driven Optimization Loop

Economic feasibility is the ultimate criterion for implementing technology. Currently, capital expenditure and operating costs remain the main obstacles.

High capital expenditure stems from high‐pressure reactors, separation units, and the catalysts themselves. Improvement directions include developing low‐cost synthesis routes (such as large‐scale production of metal–oxide supports) and reducing equipment investment per unit of capacity through process integration and heat recovery [274].

In operating expenses, electricity costs account for more than 60%, with CO_2_ separation energy consumption being the main component. Breaking the bottleneck requires a dual approach: on one hand, improving energy efficiency and single‐pass conversion rate through high current density, low voltage electrolyzers, and membrane electrode assemblies [287, 322]. On the other hand, using energy storage increases the utilization hours of renewable power, thereby reducing the average electricity price [90, 323, 324, 325].

Environmental performance is key to achieving a negative carbon emission value. A full life‐cycle carbon footprint analysis must be conducted to drive process selection, incorporating policy variables such as carbon taxes and carbon credits into the model, while reducing the carbon footprint of the entire process through metal recovery and catalyst regeneration.

#### 9.2.4 Comprehensive Solution Approach: Interdisciplinary Collaboration

In the face of the above complex challenges, a single technological breakthrough is unlikely to be effective; it is necessary to establish a cross‐disciplinary collaborative innovation platform:

##### 9.2.4.1 Materials‐Engineering Collaboration

Establish an integrated “structured catalyst development library” that combines poison resistance, high mechanical strength, and renewability, and utilize in situ characterization and machine learning to predict deactivation mechanisms, thereby accelerating formulation iterations [326].

##### 9.2.4.2 Process‐System Integration

Develop a modular microchannel‐membrane coupled experimental device to achieve integrated verification of capture, conversion, and separation. In this process, couple process intensification technologies with numerical models for simultaneous evaluation of energy consumption and cost [225, 327].

##### 9.2.4.3 Techno‐Economic and Environmental Assessment Closed Loop

Immediately conduct TEA and LCA after each experimental iteration, feeding key parameters such as carbon tax, electricity price, and catalyst lifespan back to the R&D team, dynamically adjusting catalyst formulations, reactor scales, and energy strategies to achieve cost minimization and maximize carbon reduction benefits [4, 328].

##### 9.2.4.4 Industrialization Demonstration and Policy Alignment

Collaborate with energy and chemical enterprises to establish demonstration plants with annual production capacities of tens to hundreds of tons, verifying the commercial feasibility of the technology. Actively connect with carbon trading platforms and government subsidy policies to create market‐driven demand.

Moving toward large‐scale CO_2_ conversion requires a set of interconnected system solutions: extending catalyst lifespan to over 10,000 h through structured design and intelligent regeneration [329]. Achieving efficient energy and material management through process intensification and deep integration [330, 331]. Ultimately, under the precise guidance of TEA and LCA, the technology path is continuously optimized to ensure it offers competitive benefits in both environmental and economic terms.

## 10 Technological Challenges and Future Prospects

The overarching challenges and future directions discussed in this chapter are pertinent to the entire field of catalytic CO_2_ conversion [332]. However, as emphasized throughout this review, the manifestation of these challenges and the prioritization of research solutions are intrinsically tied to the specific technological pathway—thermocatalysis or electrocatalysis [155]. The following discussion synthesizes common themes but should be interpreted with the understanding that, for instance, the “precise regulation of selectivity” or “deep integration with renewable energy” will entail fundamentally different material designs, process integrations, and economic models for a thermal methanol plant versus an electrochemical ethylene reactor.

Despite remarkable progress in catalytic CO_2_ conversion technology in recent years, transitioning from fundamental research to large‐scale industrial application still faces multiple challenges. These challenges are both the current focus of research and the direction for future development.

### 10.1 Core Scientific and Technological Challenges

For carbon dioxide electroreduction technology to achieve large‐scale and commercial applications, a series of core scientific and technological challenges still need to be overcome, and a systematic research roadmap needs to be formulated.

First, at the catalyst level, balancing cost with scalable production is the primary challenge. The current reliance on precious metals results in high costs, and the complex synthesis processes used in laboratories are challenging to scale up [333]. The breakthrough direction lies in developing catalyst systems based on abundant elements, such as iron, cobalt, nickel, and copper, and in advancing scalable processes, including continuous flow synthesis and atomic layer deposition. This approach ensures product consistency and reproducibility through online process monitoring and closed‐loop control.

Second, the precise regulation of selectivity for multi‐carbon products requires a coordinated design across multiple scales [334]. At the atomic scale, the adsorption energy of key intermediates is regulated by constructing dual‐atom sites or alloy interfaces, and local electric fields are utilized to promote C—C coupling [262, 335]. At the mesoscopic scale, structures with finite domain channels or gradient pore sizes can be designed to enrich reaction intermediates and optimize mass transfer [336]. At the system level, innovative membrane electrode assembly and reactor designs (such as gas diffusion electrode arrays) can precisely control the local reaction environment. Combined with dynamic operation strategies, such as pulsed electrolysis, they can effectively enhance reaction efficiency and inhibit catalyst deactivation [176, 305].

Furthermore, achieving deep integration with variable renewable energy is crucial to the sustainable development of technology [337, 338, 339, 340]. This requires the electrolytic system to have the capability for rapid start‐stop and wide‐load operation. Strategies to address this include designing catalyst structures that remain stable under both high and low current densities, utilizing highly conductive supports to ensure efficient electron transport, and achieving smooth energy supply‐demand matching through power management systems and energy storage devices, thereby ensuring the coordination of CO_2_ supply and electricity input [121, 341].

To systematically address the above challenges, it is recommended to follow an integrated research and development path from materials to the system.

In materials research and development, the focus is on abundant element systems such as Fe–Co and Ni–Cu, combining machine learning with high‐throughput experiments to accelerate the design and screening of high‐performance catalysts. In terms of process scale‐up, establish platforms for continuous flow pyrolysis and spray pyrolysis, promoting the stable preparation of catalysts from the gram‐scale to the kilogram‐scale. In terms of structural regulation, template methods and self‐assembly techniques are used to construct confined structures, and in situ characterization techniques are employed to monitor the reaction process in real time, guiding the multi‐layer integrated design of electrodes. In terms of system integration, build a modular electrolysis demonstration system powered by renewable energy, develop an intelligent control platform, and achieve dynamic optimization of key operating parameters.

Ultimately, through full life‐cycle assessment and economic analysis, the reliability and economic feasibility of the entire set of technologies are verified in the demonstration plant, thereby laying a solid scientific and technological foundation for establishing a sustainable pathway for CO_2_ utilization.

### 10.2 Future Research Directions and Multidisciplinary Integration

Currently, CO_2_ catalytic conversion research is undergoing a profound paradigm shift, moving from traditional “trial‐and‐error” material exploration to precise design centered on a “theory‐AI‐experiment” closed loop. This paradigm relies on large‐scale databases generated through high‐throughput experiments and computations (such as the OCx24 project, which has built a repository containing 572 samples and approximately 20,000 DFT calculation data entries), providing a solid foundation for machine learning to predict key metrics (such as adsorption energy and the Sabatier activity peak). Advanced algorithms, such as generative adversarial networks, can now increase material search efficiency by 10–100 times in multi‐step reactions. Meanwhile, cross‐scale models can couple microscopic kinetics from DFT calculations with macroscopic fluid dynamics and thermal management, enabling the evaluation of comprehensive performance at industrial‐level current densities during the material design stage. Building open AI‐experiment platforms that integrate standardized data in real time and map economic indicators will become a key infrastructure for accelerating research and development.

The application of this AI‐driven, precise design paradigm must be contextualized within the target conversion pathway [342]. The predictive models, target properties (e.g., overpotential for electrocatalysis, activation energy for thermocatalysis), and the nature of the training data (electrochemical vs. thermochemical datasets) will differ significantly between the two domains [343].

The realization of precise design heavily relies on real‐time evidence provided by in situ/operando characterization techniques. Multimodal characterization techniques (such as the combination of in situ Raman, SERS, XAS, and electrochemical microscopy) can clearly capture key intermediates on Cu‐based electrode surfaces. In contrast, liquid cell transmission electron microscopy has achieved a breakthrough in directly observing the atom‐scale dynamic reconstruction of catalysts in an electrolyte [344, 345]. These techniques reveal reaction details that were previously inaccessible. For example, spatially resolved Raman imaging has confirmed that CO_2_ reduction reactions occur only in specific alkaline microenvironments on the electrode. Integrating in situ data streams with machine learning models in real time is driving research from “post hoc analysis” toward “data‐driven mechanism learning” [194].

The choice and implementation of in situ techniques are also pathway‐specific [346]. While electrochemical techniques (like SERS under potential control) are paramount for ECO_2_R, techniques like high‐pressure operando spectroscopy or environmental TEM are more critical for probing working states in thermocatalytic reactors [347].

At the molecular level, biomimetic catalysis provides a continuous source of inspiration for designing efficient catalysts [348, 349]. By analyzing how the Rubisco enzyme in nature achieves extremely high local concentrations of CO_2_ within its “active pocket”, researchers have been able to mimic similar coordination microenvironments in artificial systems (such as metal–organic framework COFs), thereby increasing the selectivity of CO_2_ to CO conversion to over 99% [350]. A more promising direction is to construct bio‐inorganic hybrid systems by coupling natural light‐harvesting units, such as photosynthetic pigments, with artificial metal active centers, thereby opening new avenues for the light‐driven or photoelectrochemical co‐production of C_2_^+^ products [351, 352, 353].

Bio‐inspired strategies can inform both pathways. For thermocatalysis, inspiration may be drawn from metalloenzymes that catalyze hydrogenation or C—C coupling [354]. For (photo)electrocatalysis, the mimicry of photosynthetic charge separation and multi‐electron transfer is highly relevant [355].

However, any outstanding catalyst must be placed within the framework of system integration and full‐chain evaluation to demonstrate its true value. Innovative integration ideas continue to emerge. For example, using CO_2_ capture solvents directly as electrolytes enables the efficient coupling of “capture‐conversion” within the same fluid circuit, with methanol selectivity exceeding 80% [356]. Developing dual‐functional materials enables CO_2_ adsorption and catalysis to occur simultaneously on the same particle within the medium temperature range, providing a compact solution for industrial flue gas treatment [357, 358]. In this process, a comprehensive life cycle assessment of technology, economy, and environment is crucial, as it can accurately identify the key breakthroughs necessary to achieve economic feasibility [359], for example, by reducing membrane electrode impedance or optimizing energy integration schemes, thereby avoiding falling into the “high performance but high cost” dead end at the laboratory stage [360].

System integration strategies diverge sharply. Thermocatalytic pathways demand integration with hydrogen production and management of high‐grade heat, while electrocatalytic pathways require tight coupling with renewable electricity grids, power electronics, and potentially energy storage [361]. The TEA and LCA models must therefore be parameterized with pathway‐specific data (e.g., catalyst lifetime under thermal versus electrochemical stress, different balance‐of‐plant components).

Ultimately, addressing this complex challenge must rely on the establishment of interdisciplinary collaborative platforms. This requires deep integration of multiple disciplines, including computational chemistry, data science, instrument physics, process engineering, as well as economics and policy research. Specific tasks include: building a unified multi‐scale computational platform, developing specialized machine learning models, utilizing advanced characterization for real‐time monitoring, and conducting system‐level integrated design and TEA‐LCA assessments. The implementation path should systematically advance around the co‐creation of open databases, the establishment of interdisciplinary funding, the development of digital twin platforms, and the execution of industrial‐scale demonstration units.

This interdisciplinary collaboration must be channeled through a clear understanding of the two primary technological pathways [362]. Collaborative teams should be structured to tackle the distinct scientific questions (e.g., electrochemical interface science vs. high‐temperature surface chemistry) and engineering challenges (e.g., MEA design vs. fixed‐bed reactor design) inherent to each route, while also seeking synergistic learnings where possible [363].

In the future, CO_2_ catalytic conversion will no longer be an isolated “material‐hunting” process. Still, it will evolve into an innovative ecosystem characterized by four‐dimensional synergy of “materials‐mechanisms‐systems‐economics”. Only through AI‐driven, precise design, real‐time insights from in situ characterization, bio‐inspired intelligent creation, and full‐chain system integration and evaluation can the dazzling breakthroughs in the laboratory be transformed into solid, efficient, and economical industrial solutions that support global carbon neutrality goals.

## 11 Conclusion

The preceding review has systematically examined the field of CO_2_ catalytic conversion through a clarifying lens that distinguishes two primary technological pathways: thermocatalysis (driven by heat/hydrogen) and electrocatalysis (driven by electrical energy). This fundamental distinction, maintained across the discussion of materials, mechanisms, and applications, is crucial for scientific rigor and logical coherence. The various catalytic systems—heterogeneous, homogeneous, and single‐atom—were analyzed within the context of the specific pathway (thermal or electrochemical) for which they are primarily designed, highlighting their distinct design principles, performance descriptors, and challenges.

The catalytic conversion and resource utilization of CO_2_, serving as a crucial bridge connecting carbon emission control with a carbon‐circular economy, has become a strategic technological direction for addressing global climate change and promoting green and low‐carbon development. This review systematically elaborates on the latest advances in various catalytic systems for CO_2_ conversion, including heterogeneous catalysis, homogeneous catalysis, single‐atom catalysis, and electrocatalysis, revealing the core role of catalytic science in achieving the goal of "carbon balance". Through in‐depth analysis, it is evident that different catalytic systems exhibit clear complementarity and developmental trajectories: traditional heterogeneous catalysis has accumulated extensive experience in process scale‐up and industrial applications; homogeneous catalysis possesses unique advantages in mechanism elucidation and selectivity control; single‐atom catalysts, as an emerging frontier, successfully integrate the benefits of both homogeneous and heterogeneous catalysis; while electrocatalysis, due to its natural compatibility with renewable energy, demonstrates unique sustainable development potential. The parallel development of these technologies collectively forms a multi‐layered technological system for CO_2_ catalytic conversion. Current research is undergoing a significant shift from “empirical exploration” to “rational design”. Catalyst development has evolved from merely pursuing activity to precise regulation of the active site microenvironment; reaction mechanism studies have progressed from macroscopic kinetic analysis to dynamic observation at the atomic/molecular level. This transition benefits from the deep integration of advanced characterization techniques, theoretical calculations, and artificial intelligence, providing a new paradigm for catalyst design.

Nevertheless, it is also acutely aware that scaling this technology from the laboratory to practical applications still faces numerous challenges, including balancing catalyst cost and lifetime, accurately controlling the selectivity of multi‐carbon products, and integrating the system with renewable energy, all of which are critical scientific and technical issues that need to be addressed. It is imperative to recognize that these challenges manifest differently for thermocatalytic and electrocatalytic routes. For instance, selectivity control in thermocatalysis focuses on steering surface hydrogenation pathways, while in electrocatalysis it revolves around managing potential‐dependent intermediate binding and proton‐coupled electron transfer. Similarly, system integration with renewables entails coupling with green hydrogen and heat management for thermocatalysis, versus direct grid integration and load‐following operation for electrocatalysis. Solving these problems requires interdisciplinary innovation across materials science, chemistry, chemical engineering, and energy, as well as close collaboration between industry, academia, and research institutions. Looking ahead, the development of CO_2_ catalytic conversion technology will place greater emphasis on systematization and overall integration. Future studies should focus on developing efficient and stable catalysts based on earth‐abundant elements, deepening the understanding of key elementary processes such as C—C coupling, designing smart, responsive catalytic systems to adapt to fluctuating renewable energy sources, and promoting end‐to‐end innovation from catalysts to reactors and process systems. This future work must be pursued with a clear pathway‐specific strategy, advancing the unique scientific and engineering frontiers of both thermocatalytic and electrocatalytic conversion to address their respective scalability hurdles. Only through continuous breakthroughs in fundamental research and engineering innovations can catalytic technology fully realize its potential in CO_2_ resource utilization, providing solid technical support for building a sustainable, low‐carbon future.

## Funding

This study was supported by Ministry of Education and Science of the Republic of Kazakhstan (grant BR24992964).

## Conflicts of Interest

The authors declare no conflicts of interest.

## Data Availability

The data that support the findings of this study are available on request from the corresponding author. The data are not publicly available due to privacy or ethical restrictions.
